# SWI/SNF complexes govern ontology-specific transcription factor function in MYC-subtype atypical teratoid rhabdoid tumor

**DOI:** 10.1093/neuonc/noaf081

**Published:** 2025-03-23

**Authors:** Cody L Nesvick, Liang Zhang, Yuqian Yan, Alexander Q Wixom, Feda H Hamdan, Jizhi Ge, Jacob B Anderson, Alexandre Gaspar-Maia, Steven A Johnsen, David J Daniels

**Affiliations:** Department of Neurological Surgery, Mayo Clinic, Rochester, Minnesota, USA; Department of Neurological Surgery, Mayo Clinic, Rochester, Minnesota, USA; Department of Neurological Surgery, Mayo Clinic, Rochester, Minnesota, USA; Department of Gastroenterology and Hepatology, Mayo Clinic, Rochester, Minnesota, USA; Department of Gastroenterology and Hepatology, Mayo Clinic, Rochester, Minnesota, USA; Department of Neurological Surgery, Mayo Clinic, Rochester, Minnesota, USA; Department of Molecular Pharmacology and Experimental Therapeutics, Rochester, Minnesota, USA and Mayo Clinic Graduate School of Biomedical Sciences, Mayo Clinic Alix School of Medicine, and Mayo Clinic Medical Scientist Training Program, Rochester, Minnesota, USA; Department of Laboratory Medicine and Pathology, Mayo Clinic, Rochester, Minnesota, USA; Epigenomics Program, Center for Individualized Medicine, Mayo Clinic, Rochester, Minnesota, USA; Robert Bosch Center for Tumor Diseases, Stuttgart, Germany; Molecular Pharmacology and Experimental Therapeutics Program, Mayo Clinic, Rochester, Minnesota, USA; Department of Neurological Surgery, Mayo Clinic, Rochester, Minnesota, USA

**Keywords:** ATRT, chromatin, epigenetics, rhabdoid tumor, transcription factors

## Abstract

**Background:**

Atypical teratoid rhabdoid tumor (ATRT) is a deadly central nervous system embryonal tumor caused by loss of SMARCB1, a core subunit of SWItch/Sucrose Non-Fermentable (SWI/SNF) chromatin remodeling complexes. SMARCB1-deficient cancers are defined by loss of cell differentiation-associated enhancers, but how SWI/SNF interacts with other arbiters of cell differentiation (specifically lineage-specific transcription factors [TFs]) remains poorly understood.

**Methods:**

We leveraged a multi-omics approach, patient-derived ATRT cells, and patient-derived orthotopic xenografts to investigate the interplay of SWI/SNF with lineage-specific TFs in a clinically relevant setting.

**Results:**

We observe that an activating protein 1 (AP-1)-dependent transcriptional regulatory network is lost in ATRT, and AP-1 and lineage-specific TFs TEAD1 and ZIC2 require SMARCB1 for enhancer binding. SMARCB1-dependent SWI/SNF integrates transcriptional functions of lineage-specific TFs into a core regulatory circuit that depends on the AP-1 subunit c-JUN, whose expression is determined by a SMARCB1-dependent super-enhancer that is lost in ATRT-MYC. In the absence of SMARCB1, lineage-specific TFs are sequestered to promoters, where they maintain core transcriptional programs necessary for cell survival. Targeting residual, promoter-proximal TF activity by a protein degrader of the SWI/SNF ATPase SMARCA4 or small-molecule inhibitors that indirectly inhibit AP-1 and TEAD activity abrogates expression of these networks, reducing cell viability in vitro and prolonging survival in an orthotopic patient-derived xenograft model.

**Conclusions:**

These results demonstrate SWI/SNF complexes are critical for lineage-specific TF binding and activity at both promoters and enhancers. In the context of ATRT, these findings reveal a previously underappreciated therapeutic vulnerability in targeting residual promoter-proximal TF function in ATRT.

## Graphical Abstract

**Graphical Abstract:**
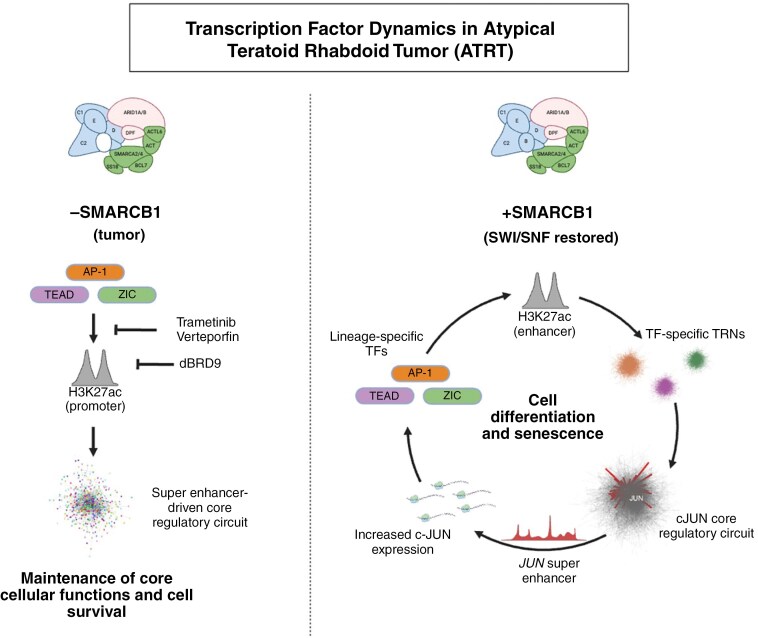

**Key Points**

1. SMARCB1 is necessary for recruitment of lineage-specific transcription factors (TFs) to enhancers.
2. In ATRT, SMARCB1 loss sequesters lineage-specific TFs to promoters.
3. Residual SWI/SNF and TF activity in ATRT can be therapeutically targeted.

**Importance of the Study**

Atypical teratoid rhabdoid tumor (ATRT) is an embryonal tumor of the nervous system that is almost always caused by loss of SMARCB1, a subunit of the SWI/SNF chromatin remodeling complex. In the field of chromatin biology, it has been debated how SWI/SNF interacts with lineage-specific transcription factors (TFs) to activate enhancers that are necessary for cell differentiation, which is a key translational question for clinical oncology. Using patient-derived cell lines, we demonstrate SMARCB1 is necessary for TF recruitment to enhancer loci, and SWI/SNF integrates TF-associated enhancers into a core regulatory circuit that is lost in ATRT. In the absence of SMARCB1, lineage-specific TFs are sequestered to promoters of genes necessary for cell survival, and expression of these transcription networks can be targeted using clinically available protein degraders and small-molecule inhibitors. These results are a key step in translating our evolving understanding of SWI/SNF biology into clinical practice.

Atypical teratoid rhabdoid tumor (ATRT) is a rare central nervous system (CNS) tumor of early childhood. The median overall survival is less than 1 year, and while aggressive multi-modal treatment regimens have improved prognosis, most patients die of refractory or recurrent disease, and long-term survivors frequently suffer significant treatment-related morbidity.^1,2^ A prominent feature of ATRT is its genomic stability: the only recurrent genomic abnormality in most ATRTs is bi-allelic loss or inactivation of *SMARCB1*, which codes for a core subunit of the SWI/SNF (also known as BRG1-/BRM1-associated factor, or BAF) chromatin remodeling complex.^3–5^

SWI/SNF is an evolutionarily conserved complex that is essential for enhancer selection throughout development (reviewed in Kadoch and Crabtree^6^), and mutations in a SWI/SNF subunit are found in 20-25% of all human cancers.^7^ In ATRT and other SMARCB1-deficient rhabdoid tumors, SMARCB1 loss results in genome-wide loss of enhancers necessary for normal development.^8–10^ While SWI/SNF complexes have been proposed to cooperate with lineage-specific transcription factors (TFs) to define enhancers,^11^ the mechanisms by which this occurs are undefined, and how this might contribute to ATRT pathogenesis is unknown.

To determine the mechanisms underpinning enhancer reprogramming in ATRT, we leveraged a multi-omics approach that utilizes both patient-derived cell lines and molecular data acquired from ATRT specimens. In addition to loss of predicted promoter-distal roles for activating protein 1 (AP-1) in this setting, we observe SMARCB1 restoration results in activation of enhancers associated with lineage-specific TFs TEAD1 and ZIC2 that converge on a c-JUN-dependent core regulatory circuitry (CRC) that is specific to ATRT-MYC, which is characterized by homozygous deletion of *SMARCB1.* Moreover, in the absence of SMARCB1, these TFs are largely sequestered to the promoters of metabolic genes whose expression remains dependent on residual SWI/SNF. Taken together, our results highlight a central role for SWI/SNF in dictating the genomic distribution and epigenetic function of lineage-specific TFs and offer insights into how loss of SWI/SNF function might contribute to ATRT pathogenesis.

## Methods

### Cell Culture and Western Blot

Details for methodology pertaining to cell culture; Western blot; RNA extraction, library preparation, and sequencing; and ChIP-Seq sample preparation, library preparation, and sequencing can be found in the Supplementary Information file.

### RNA-Seq Data Analysis

All FASTQ files underwent quality control assessment using FASTQC (https://www.bioinformatics.babraham.ac.uk/projects/fastqc/). Proximal read trimming was performed to ensure an average Phred score of at least 32 for read length under study. Trimmed reads were then mapped to hg38.p13 and counted using STAR v 2.7.3a (--quantMode GeneCounts).^12^ Normalized gene counts and differential gene expression analysis were performed using DESeq2^13^ with a false discovery rate (FDR) < 0.05 defining a statistically significant difference in gene expression. Enrichment for gene expression was performed using Gene Set Enrichment Analysis (GSEA).^14^ Gene ontology (GO) analyses were performed using the Gene Ontology Resource (https://geneontology.org/) and visualized using the Cytoscape^15^ (v3.8.2) ClueGO plugin (v2.5.8). For visualization, ClueGO settings were: *P* < .00001 for GO term enrichment, term fusion ON, min # 10 genes for pathway, and Kappa 0.4. Principal component analysis (PCA) was used to assess for inter-sample variation; PCA plots for RNA-Seq data generated in this study, along with Venn diagrams for overlapping up- and downregulated genes across cell lines, can be found in Supplementary Figure S1.

### Bioinformatics Analysis of ChIP-Seq Data

ChIP-Seq files underwent rigorous quality control as described above. Reads were mapped to hg38.p13 using bowtie2 v2.3.3.1 (–very-sensitive).^16^ Read pileups and BigWig matrices were visualized with DeepTools,^17^ removing duplicates and hg38 ENCODE blacklisted regions bamCoverage. Peak tracings shown were generated using pyGenomeTracks.^18^

ChIP-Seq peaks were called relative to condition-equivalent input using MACS2.^19^ For TF and SWI/SNF ChIPs, a hard-masked genome build was used to prevent arbitrary assignment of reads to repetitive genomic elements. For TF and SWI/SNF ChIPs, narrow peaks were called using an FDR cutoff of 10^−3^. Motif enrichment analysis was performed using HOMER^20^ (de novo motif enrichment function). For peak overlaps, peaks were extended bilaterally 150 bp from the peak center (ie, slightly more than 1 nucleosomal unit) to detect overlap with candidate cooperative TFs. For histone modifications, broad peaks were called using an FDR cutoff of 10^−1^. Default ROSE^21,22^ parameters were used to call enhancers from H3K27ac peaks, and unique constituent enhancers were defined by subtracting H3K27ac peaks of the SMARCB1-absent condition from the SMARCB1-restored condition using BEDTools.^23^ Active promoters were defined by H3K4me3 occupancy by ChIP-Seq.

**Figure 1.**
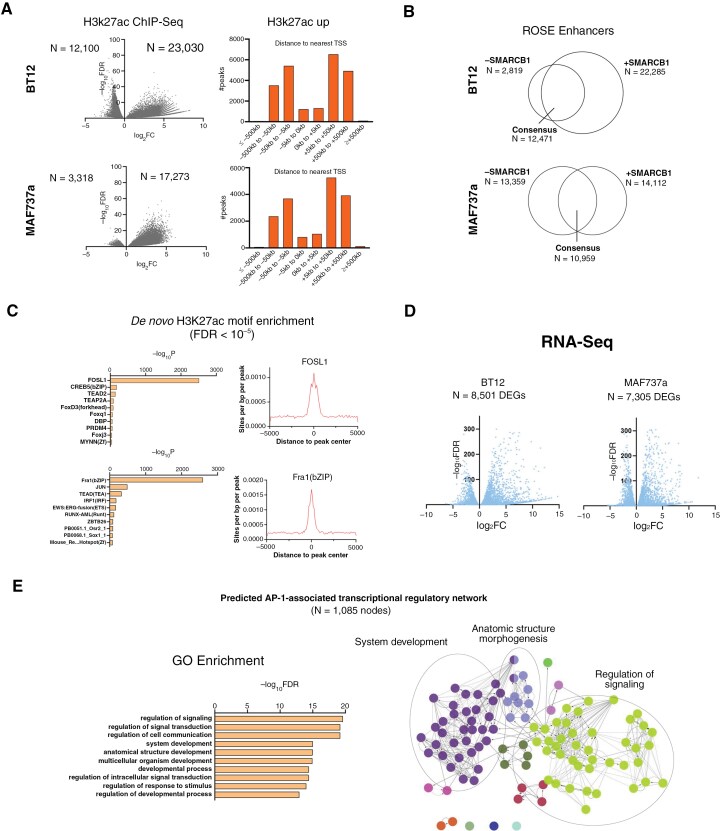
**Enhancer and transcriptional landscapes in SMARCB1-absent and -restored ATRT cells.** (**A**) SMARCB1 restoration remodels enhancers. Volcano plots demonstrate differentially bound H3K27ac loci in SMARCB1-restored vs. -absent ATRT cells (FDR < 0.05, left). Accompanying histograms detail the genomic distribution of gained H3K27ac peaks relative to the nearest transcription start site (TSS) (right) in BT12 and MAF737a cells. (**B**) Enhancer dynamics. Relative overlap in active enhancers called by Rank Ordering of Super-Enhancers (ROSE) highlights altered enhancer loci in SMARCB1-absent and -restored cells. (**C**) Transcription factor motif enrichment. De novo motif enrichment analyses using HOMER of constituent H3K27ac broad peaks contributing to gained enhancers in SMARCB1-restored cells (broad peak FDR < 10^−5^), identifying potential transcriptional regulators mediating enhancer activation. (**D**) Transcriptional consequences of SMARCB1 restoration. Volcano plots illustrating differentially expressed genes (DEGs) between SMARCB1-absent and restored BT12 and MAF737a cells (FDR < 0.05), highlighting transcriptional reprogramming. (**E**) Functional enrichment of SMARCB1-dependent transcriptional circuitry. Gene ontology (GO) enrichment of predicted AP-1 associated transcriptional regulatory circuitry in BT12 cells, linked to de novo enhancers using the single-nearest gene method. The most highly enriched terms and key ontological families are highlighted.

Gene-region interactions in exploratory analyses (Figure 1) were performed by assigning enhancer peaks to the single-nearest coding region of the genome. For more granular analyses of TF-associated enhancers (all other analyses), enhancer peaks were overlapped with high-confidence enhancer targets using GeneHancer (Double Elite setting).^24^ Transcriptional regulatory networks were defined as predicted enhancer-gene interactions whose target genes demonstrated increased expression by RNA-Seq.

### Patient-Derived Xenografts (PDXs)

BT12 and BT16 cell lines were transduced with a lentiviral vector expressing green fluorescent protein (GFP)-luciferase. All animals were handled in accordance with IACUC guidelines. The Mayo Clinic Institutional Committee for Animal Research approved all experiments. In brief, 300,000 cells were placed in single-cell suspension in 3 µl media and then stereotactically injected into the right frontal lobe of 6- to 7-week-old female athymic nude mice (Hsd:athymic Nude-Foxn1nu mice, Envigo). Stereotactic injections were performed using a 26-gauge syringe (Hamilton). Initial tumor growth was detected at 7 days using In Vivo Imaging System (IVIS) bioluminescence imaging (Xenogen). Following tumor detection, animals were randomized to treatment groups by weight. Control animals were treated with 3 µl dimethyl sulfoxide (DMSO) solution (10% DMSO, 90% phosphate-buffered saline [PBS]) intraperitoneally 3 times weekly. Experimental animals were treated with trametinib, 0.5 mg/kg, by oral gavage 5 days per week. Animals were monitored daily and followed until death, moribund state, or severe neurologic deficit. Survival analyses were performed using the Mantel-Cox log-rank test.

### Data Availability

Sequencing data for the experiments presented in this study will be made available upon reasonable request to the corresponding author.

### ATRT Patient Samples

RNA-Seq data from primary ATRT specimens were obtained from St. Jude Children’s Research Hospital (SJCRH). Analysis of ATRT patient specimen RNA-Seq data was performed using pre-processed counts files mapped to hg38 according to the SJCRH pipeline. Only specimens obtained at diagnosis (“D1,” *N* = 27) were initially analyzed. Counts from all subgroups were compiled into a normalized counts table using DESeq2.^13^ Visual inspection of PCA analysis demonstrated *N* = 2 samples did not readily classify into 1 molecular subgroup with the current sample size; for accurate subgrouping, these were excluded from downstream analysis. Molecular subgroup was defined by clustering on PCA analysis and expression of highly specific markers of ATRT-SHH (*ASCL1*), ATRT-TYR (*TYR*, *MITF*), and ATRT-MYC (*MYC*, *HOXC* genes).^25–27^ Differential gene expression and GSEA were performed as described above.

### Ethics Statement

No institutional review board approval was required for the studies described. RNA-Seq data for human tumors was acquired via data transfer agreement between Mayo Clinic and SJCRH. No private health information was shared as part of this agreement. The authors have no conflicts of interest to declare.

## Results

### A Putative AP-1-Associated Developmental Transcriptional Regulatory Network Is Lost in ATRT

To study how SMARCB1 loss alters the enhancer landscape, we restored SMARCB1 expression in patient-derived ATRT cell lines and performed chromatin immunoprecipitation with sequencing (ChIP-Seq) for histone post-translational modifications (PTMs) marking enhancers (H3K27ac, H3K4me1), promoters (H3K4me3) and polycomb-repressed regions (H3K27me3). Consistent with prior studies,^10,28,29^ SMARCB1 restoration led to proliferative arrest across cell lines of multiple molecular subtypes of ATRT (Supplementary Figure S2A-D). Mechanistically, transcriptomic analyses confirmed enrichment of cell cycle-associated transcriptional targets and senescence-associated genes in SMARCB1-absent and -restored cells, respectively (Supplementary Figure S2E). SMARCB1 restoration had a substantial impact on the genome-wide distribution of the activating mark H3K27ac, with numerous gained peaks corresponding to promoter-distal loci consistent with enhancers (Figure 1A). Indeed, the vast majority of H3K27ac peaks were identified as bona fide enhancers by Rank Ordering of Super-Enhancers (ROSE^21^) (Figure 1B). To elucidate possible TFs that may play a role at SWI/SNF-dependent enhancers, we performed motif enrichment analysis of H3K27ac enhancer peaks defined with high confidence (FDR < 10^−5^). Gained enhancer peaks demonstrated robust central enrichment of the AP-1 consensus motif, indicating a central role for AP-1 at SWI/SNF-dependent enhancers (Figure 1C).

The transcriptomic impact of the SMARCB1-dependent enhancer landscape was then quantified using RNA-Seq. Differential gene expression analysis demonstrated a dramatic transcriptomic shift following SMARCB1 restoration, further reinforcing the fundamental role of SWI/SNF composition in defining transcriptional identity of the cell (Figure 1D). We next sought to relate these transcriptional changes to specific enhancers containing the AP-1 motif. Leveraging proximity-based analysis, an AP-1-associated transcriptional regulatory network (TRN) was defined as consisting of upregulated genes predicted to be targets of AP-1-dependent enhancers. GO analysis of this network demonstrated significant enrichment for numerous terms associated with development and anatomic structure morphogenesis (Figure 1E), implicating AP-1 in the developmental regulatory circuitry that is lost in ATRT.

### SMARCB1 Is Necessary for the Association of Lineage-Specific TFs with Enhancer Loci

AP-1 is both a classic cancer- and enhancer-associated TF that is increasingly appreciated as a possible pioneer factor for enhancers and physically associates with SWI/SNF in some settings.^11,30^ AP-1 subunits accumulate promiscuously at enhancers,^31^ cooperate with lineage-specific TFs at cell type-specific enhancers^11,32^ and may guide SWI/SNF-dependent 3-dimensional chromatin organization.^33,34^ However, the degree to which SWI/SNF itself is necessary for enhancer-associated functions of AP-1 and lineage-specific TFs has not been thoroughly investigated.

**Figure 2.**
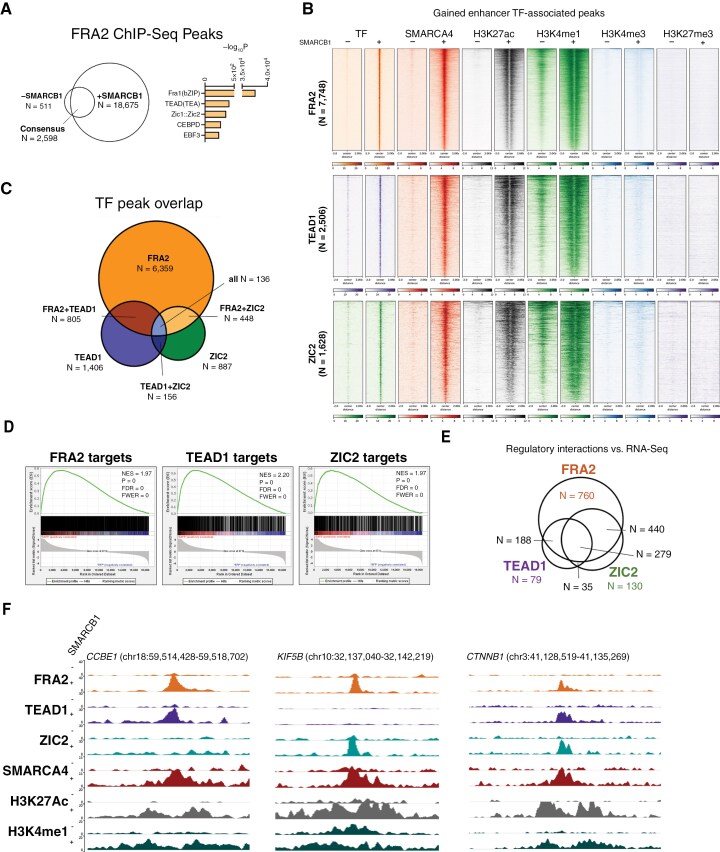
**SMARCB1 restoration redirects lineage-specific TFs to activated enhancers.** (**A**) SMARCB1-dependent redistribution of FRA2. FRA2 ChIP-Seq peak overlap analysis in BT12 in SMARCB1-absent and -restored BT12 cells. De novo motif enrichment analysis in the union peak set highlights additional TF families that may co-bind with FRA2. (**B**) TF engagement at SMARCB1-dependent enhancers. Read pileups of SMARCB1-dependent active enhancers associated with FRA2, TEAD1, and ZIC2, demonstrating their recruitment to enhancer loci only after SMARCB1 restoration. (**C**) TF occupancy at gained enhancers. Relative overlap of TF peaks at gained active enhancers following SMARCB1 restoration, illustrating significant but limited coordination in TF recruitment. (**D**) Transcriptional impact of TF-associated enhancers. Gene set enrichment analysis (GSEA) of TF-associated enhancer target gene expression assessed by RNA-Seq. Target genes were assigned to enhancers using a curated library of regulatory elements (GeneHancer). (**E**) Convergence of TF-regulated transcriptional programs. Venn diagram illustrating overlap in TF-associated enhancer target genes upregulated following SMARCB1 restoration, highlighting cooperation in regulatory circuits. (**F**) Local TF cooperation at developmentally relevant enhancers. Representative genomic loci demonstrating TF co-binding at developmentally pertinent enhancers.

To determine how SMARCB1 influences lineage-specific TF function in the context of ATRT, we first performed ChIP-Seq to examine the genome-wide occupancy of an AP-1 subunit in SMARCB1-absent and -restored ATRT cell lines. Among AP-1 subunits, FRA2 (also known as FOSL2) was chosen as a model subunit owing to high expression across models at the transcript and protein level (Supplementary Figure S3). SMARCB1 restoration was associated with a net gain of FRA2 peaks (Figure 2A; Supplementary Figure S4A). The AP-1 family is expressed in numerous cell lineages throughout development and is predicted to cooperate with lineage-specific TFs at key developmental enhancers.^11^ Given our interest in studying the interplay of SWI/SNF-dependent enhancers, TFs, and transcription, we next sought to probe this possibility before further study. Motif enrichment analysis of FRA2 peaks demonstrated significant enrichment for the transcriptional enhancer factor (TEF/TEA) domain (TEAD)-containing TF family as well as the ZIC TF family in 1 model (Figure 2A; Supplementary Figure S4A). The TEAD family of TFs has been shown to be necessary for the normal cellular function of multiple cell lineages, including the placenta, trophoblast, smooth muscle, and neural lineages.^35^ ZIC TFs are well-established effectors that are critical for neural differentiation, particularly hindbrain formation.^36–42^ The combination of shared motif enrichment, plausible biological cooperation, and predicted pioneering function of these TFs led us to hypothesize that SMARCB1-dependent enhancer loci could be predicted by TF binding sites.

To test this hypothesis, we performed ChIP-Seq on TEAD1, ZIC2, and the shared SWI/SNF ATPase subunit SMARCA4 (BRG1) in SMARCB1-absent and -restored cells. TEAD1 and ZIC2 were chosen as representative TF family members owing to their reliable expression across ATRT models (Supplementary Figure S3). Integrated epigenomic analysis demonstrated FRA2, TEAD1, and ZIC2 loci were defined by 4 distinct chromatin states: active promoters (H3K4me3+), active SMARCB1-independent enhancers (H3K4me1+, H3K27ac+ in SMARCB1-absent cells), active SMARCB1-dependent enhancers (H3K4me1+, H3K27ac+ in SMARCB1-restored cells), and poised enhancers (H3K4me1+ only) (Supplementary Figure S5). Contrary to our hypothesis, close analysis of these associations revealed many, if not most, TF-associated active enhancers were specific to the SMARCB1-restored condition (Figure 2B and Supplementary Figure S4B). Additionally, although peak overlap between FRA2, TEAD1, and ZIC2 peaks was significantly greater than expected by chance (Fisher’s *P* ~ 0 for all pairwise comparisons), most TEAD1 and ZIC2 peaks associated with SMARCB1-dependent enhancers did not overlap with FRA2-binding sites (Figure 2C; Supplementary Figure S4C). Taken together, these findings demonstrate not only that SMARCB1 is necessary for the association of lineage-specific TFs with active enhancers, but also that AP-1 cooperativity with other TF families may be less than predicted by prior *in silico* analyses.

In light of this unexpected finding, we next sought to further identify enhancer-dependent TRNs associated with each TF under study. To define TF-associated TRNs, epigenomic and transcriptomic data were integrated using a curated library of annotated regulatory elements^24^ to assign predicted target genes to SMARCB1-dependent, TF-associated enhancers. Bona fide gene targets were identified by RNA-Seq. Predicted FRA2, TEAD1, and ZIC2 target gene sets were indeed highly enriched in SMARCB1-restored cells (Figure 2D; Supplementary Figure S4D). Intriguingly, the vast majority of TEAD1 and ZIC2 enhancer targets were also targeted by FRA2, indicating that SMARCB1 and SWI/SNF may serve to integrate TF-associated enhancers into a shared TRN (Figure 2E; Supplementary Figure S4E). Indeed, upregulated target genes of TF-associated enhancers were significantly enriched for development- and differentiation-associated ontological terms, supporting the biological relevance of this assertion (Supplementary Figure S6). Exemplary enhancers targeting developmentally pertinent genes *CCBE1*, *KIF5B*, and *CTNNB1* are shown in Figure 2F.

To further validate these findings, we repeated key experiments in a patient-derived ATRT-MYC stem-cell model (DJD29) that accurately retains many unique features of this molecular subtype that are frequently lost in in vitro models (ie, *HOX* cluster activation). Consistent with the other 2 models, SMARCB1 restoration redirected TEAD1 and ZIC2 to SMARCB1-dependent enhancers (Supplementary Figure S7A-C). TF-associated enhancer target genes were highly enriched in SMARCB1-restored cells (Supplementary Figure S7D), and substantial overlap in ZIC2 and TEAD1 target genes was observed (Supplementary Figure S7E). These findings further underscore the fundamental role of SMARCB1 in defining the biological function of lineage-specific TFs at enhancers.

### SMARCB1 Restoration Mimics Signal-Dependent Enhancer Selection via JUN Upregulation

**Figure 3.**
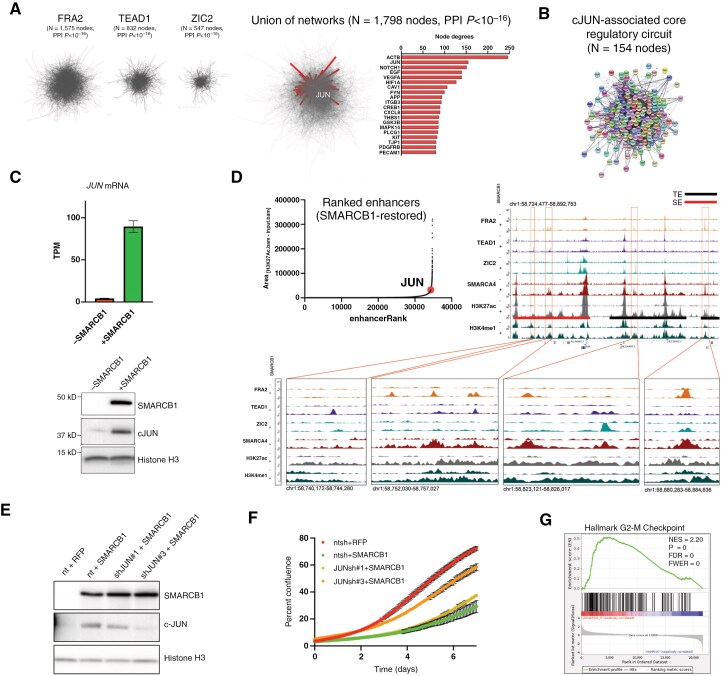
**Transcription factor-mediated interactions define a conserved c-JUN-oriented core regulatory circuitry.** (**A**) Network cooperativity of TF-associated enhancers. Protein-protein interaction (PPI) network analysis using upregulated target genes of enhancers associated with FRA2, TEAD1, and ZIC2-associated enhancers as input. The right panel highlights the union of networks, with the most highly integrated nodes further emphasized. (**B**) c-JUN core regulatory circuitry. Visualization of single-nearest neighbors of c-JUN, identifying the key upregulated partners of c-JUN that make up the c-JUN core regulatory circuitry (CRC). (**C**) c-JUN expression following SMARCB1 restoration. *JUN* mRNA and c-JUN protein expression assessed by RNA-Seq and Western blot, respectively. Significance was assessed using the unpaired 2-tailed Student’s *t*-test; ***P < .001. (**D**) Super-enhancer regulation of c-JUN. Ranked enhancer analysis (ROSE) of SMARCB1-restored cells highlighting the *JUN* super-enhancer. The y-axis in panel F represents the average difference in area (in arbitrary units) within the H3K27ac ChIP-Seq peaks and inputs at each locus. The *JUN* genomic neighborhood is shown for illustration of the associated super-enhancer. (**E**) *JUN* knockdown in SMARCB1-restored cells. Western blot of ATRT cells following SMARCB1 restoration and transduction with either nontargeting (NT) or *JUN* shRNA, assessing the impact of *JUN* depletion. (**F**) c-JUN-dependent cell proliferation. Incucyte proliferation analysis of cells analyzed in E, quantifying the phenotypic impact of *JUN* knockdown. (**G**) Transcriptional impact of *JUN* loss. Gene set enrichment analysis of SMARCB1-restored cells following *JUN* knockdown, with gene set identifiers shown. BT12 cells were used for all analyses in this figure.

Following the surprising observations that SMARCB1-associated SWI/SNF both recruits TFs to enhancer loci and integrates transcriptional targets, we next sought to assess the biological importance of these observations by leveraging a computational approach. To accomplish this, an annotated library of predicted and experimentally established protein-protein interactions (PPI)^43^ was used to integrate TF-associated enhancer target genes into functional cell biological networks. Strikingly, each TF-associated TRN contained significantly more interactions than would be expected by chance (PPI *P* < 10^−16^, Figure 3A; Supplementary Figure S8A), supporting the concept that SMARCB1-dependent enhancers serve to integrate TF target genes into ontologically meaningful networks. Surprisingly, the second-most highly integrated node in the shared TF-associated network (ie, direct interactions with other network members) was the AP-1 subunit c-JUN (Figure 3A; Supplementary Figure S8A). The c-JUN core regulatory circuit (CRC), consisting of proteins predicted to directly interact with c-JUN, demonstrated a diverse array of proteins critical for a variety of developmental processes, including EGF, FGF, NOTCH2, TGFBR2, and NEDD4 (Figure 3B; Supplementary Figure S8B). Furthermore, significant overlap was observed in the c-JUN CRC between models, highlighting conservation of the c-JUN CRC in the SMARCB1-dependent phenotype lost in ATRT (Supplementary Figure S8C).

Given the centrality of c-JUN in this model, we next assessed *JUN* mRNA and c-JUN protein expression. *JUN* transcript levels were significantly increased in SMARCB1-restored cells, and this translated to increased c-JUN protein expression (Figure 3C; Supplementary Figure S8D). Chromatin state analysis of the *JUN* neighborhood confirmed an active state of the *JUN* promoter in both conditions, but SMARCB1 restoration led to activation of a super-enhancer (SE) assigned to *JUN* specific to SMARCB1-restored cells (Figure 3D; Supplementary Figure S8E). Taken together with our observation that c-JUN is central to SMARCB1-dependent regulatory circuitry, we hypothesized that c-JUN specifically is necessary for the transcriptional effects of SMARCB1 restoration. To test this, we knocked down *JUN* using small hairpin RNA (shRNA) in SMARCB1-restored cells (Figure 3E), and this was sufficient to reverse the proliferative arrest induced by SMARCB1 in a dose-dependent fashion (Figure 3F). We thus hypothesized that c-JUN is necessary for execution of the SMARCB1-dependent senescent transcriptional program. Indeed, GSEA of RNA from SMARCB1-restored cells confirmed enrichment of genes associated with cell-cycle arrest in *JUN-*knockdown cells compared to nontargeting controls (Figure 3G). Together, these data demonstrate that the AP-1 subunit c-JUN is the key effector linking lineage-specific TF-associated enhancers with the senescent phenotype observed in SMARCB1-restored ATRT cells.

AP-1 has been proposed as a master regulator of multiple enhancers associated with cell-state transitions including pluripotency^44^ and senescence,^45^ raising the possibility that SMARCB1-induced enhancer reprogramming may simply reflect increased expression of c-JUN following SMARCB1 restoration. To determine whether c-JUN expression alone is sufficient for enhancer gain in the absence of SMARCB1, we constitutively over-expressed c-JUN and performed ChIP-Seq for H3K27ac and H3K4me1 in SMARCB1-absent cells (Supplementary Figure S9A). While c-JUN over-expression alone had relatively modest effects on cell proliferation (Supplementary Figure S9B), this was not sufficient to activate AP-1-associated enhancers, indicating c-JUN upregulation requires the epigenetic context of SMARCB1 restoration to induce a senescent phenotype (Supplementary Figure S9C).

### Loss of c-JUN Expression and the c-JUN-Dependent Transcriptional Regulatory Network Is Specific to ATRT-MYC

**Figure 4.**
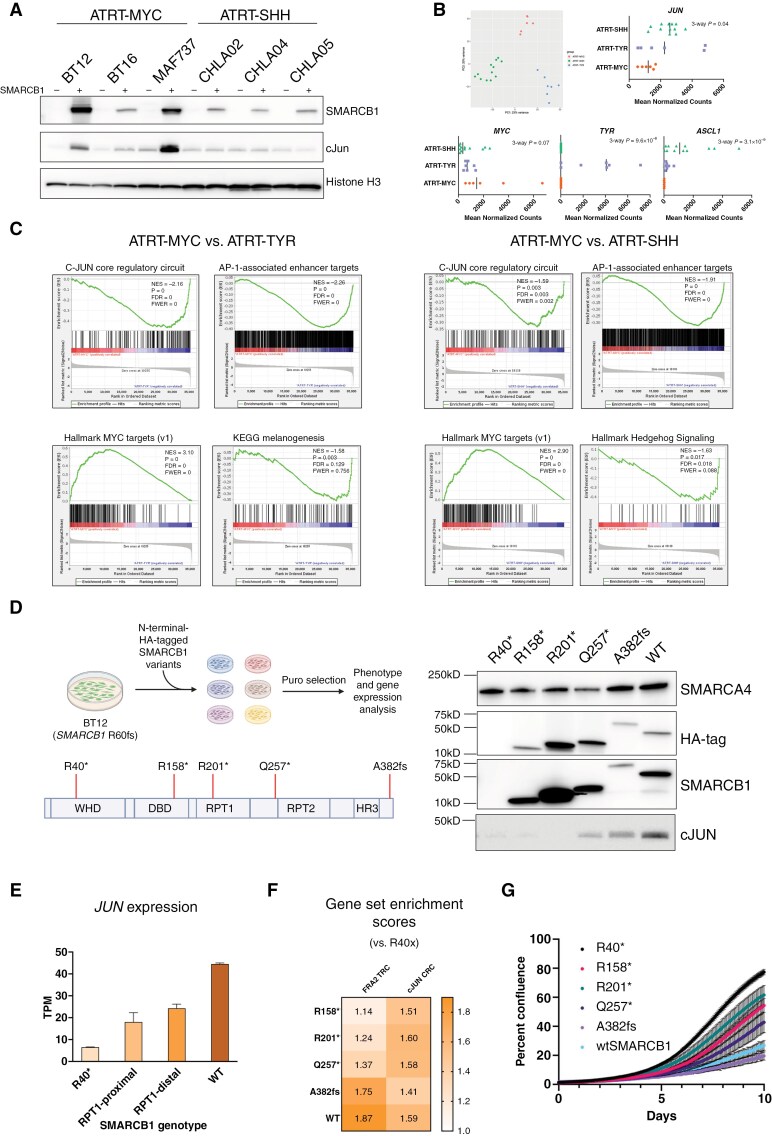
**Loss of AP-1-associated enhancers and the c-JUN core regulatory circuitry are specific to ATRT-MYC.** (**A**) c-JUN expression across cell lines of multiple ATRT subtypes. Western blot of c-JUN expression in ATRT-MYC and ATRT-SHH cell lines following SMARCB1 restoration, illustrating subtype-specific differences in c-JUN regulation. (**B**) Differential JUN expression across ATRT subtypes. Principal component analysis (PCA) of RNA-Seq data from 25 untreated ATRT specimens demonstrating 3 molecular subgroups, with expression of key subgroup markers and *JUN* expression shown. (**C**) Subtype-specific enrichment of c-JUN-associated transcriptional programs. Gene set enrichment analysis of subgroup-relevant genes, c-JUN CRC component genes, and the AP-1-associated TRN in ATRT patient specimens. (**D**) Patient-specific SMARCB1 mutants. Schematic for experimental design utilizing patient-specific *SMARCB1* mutants (right). Expression of HA-tagged SMARCB1 variants was confirmed by Western blot. (**E**) *JUN* transcriptional response to SMARCB1 variants. *JUN* mRNA expression by RNA-Seq in *SMARCB1* variant subgroups, highlighting variant-specific impacts on *JUN* expression. (**F**) Transcriptional impact of SMARCB1 variant expression. Gene set enrichment for the c-JUN CRC and AP-1 TRN across *SMARCB1* variant-expressing cells, illustrating their impacts on network expression. (**G**) Proliferative effects of SMARCB1 variants. Incucyte proliferation assay of BT12 cells transduced with selected *SMARCB1* variants, demonstrating their impact on proliferative dynamics.

While most ATRTs share the feature of SMARCB1 loss and genomic stability, at least 3 major molecular subgroups of SMARCB1-altered ATRT exist (ATRT-MYC, ATRT-TYR, and ATRT-SHH).^25,26^ As the cell lines used in the above analyses transcriptionally classify as ATRT-MYC (see Methods), we further assessed the effect of SMARCB1 restoration on c-JUN expression in a panel of ATRT-MYC and ATRT-SHH cell lines. ATRT-TYR cells were not tested due to lack of reliable in vitro models. Interestingly, all 3 ATRT-MYC cell lines demonstrated increased c-JUN expression following SMARCB1 restoration, which was observed in none of the ATRT-SHH cell lines (Figure 4A).

Given these intriguing findings, we hypothesized that loss of c-JUN expression, AP-1-associated enhancers, and the c-JUN-oriented CRC are specific findings of the ATRT-MYC subtype. To test this, we turned to transcriptomic data obtained in ATRT specimens. PCA demonstrated clustering of specimens into 3 distinct subgroups (Figure 4B).^25,26^ In line with our experimental observations, *JUN* expression was significantly downregulated in ATRT-MYC compared to ATRT-TYR and -SHH specimens (Figure 4B). Expression of centrally important genes *MYC*, *TYR*, and *ASCL1* demonstrated subtype-specific enrichment, consistent with prior work (Figure 4B). Furthermore, in addition to enrichment of developmental pathways and gene sets that are known to distinguish ATRT subgroups, we observed significant depletion of genes associated with the c-JUN CRC and predicted AP-1-dependent TRN in ATRT-MYC specimens compared to ATRT-TYR and ATRT-SHH (Figure 4C).

To further interrogate the link between ATRT-MYC and loss of AP-1-associated regulatory circuitry, we leveraged an experimental biological approach by expressing pathogenic SMARCB1 variants in the background of functionally SMARCB1-null ATRT (Figure 4D). ATRT-MYC is in part distinguished from ATRT-TYR by *SMARCB1* genotype, with whole-gene *SMARCB1* deletions being more common in ATRT-MYC, and more focal *SMARCB1* alterations more common in ATRT-TYR.^25,26^ C-terminal-mutant SMARCB1 is predicted to integrate with SWI/SNF but leads to an incompletely functional chromatin remodeling complex.^46^ We engineered BT12 cells to express mutant SMARCB1 proteins spanning each major functional domain of SMARCB1 protein and assessed the impact on c-JUN expression and AP-1-associated networks by Western blot and RNA-Seq, respectively. RPT-1-distal SMARCB1 mutants (Q257* and A382fs) led to increased expression of c-JUN by Western blot but without reaching levels achieved by full-length, wildtype SMARCB1 (Figure 4D). This stepwise increase in expression with increasing *SMARCB1* transcript length was also observed at the RNA level (Figure 4E), suggesting the molecular functions of SMARCB1 that are lost in ATRT-MYC are necessary for c-JUN expression. GSEA likewise demonstrated graded enrichment for the AP-1 TRN and c-JUN CRC (Figure 4F), and ATRT-TYR-type *SMARCB1* mutants were associated with a more profound effect on cell viability (Figure 4G). Taken together, these data illustrate that loss of c-JUN expression and the associated regulatory circuitry is a specific feature of ATRT-MYC.

### SWI/SNF Composition Is Necessary for the Epigenomic Distribution and Function of Lineage-Specific TFs

**Figure 5.**
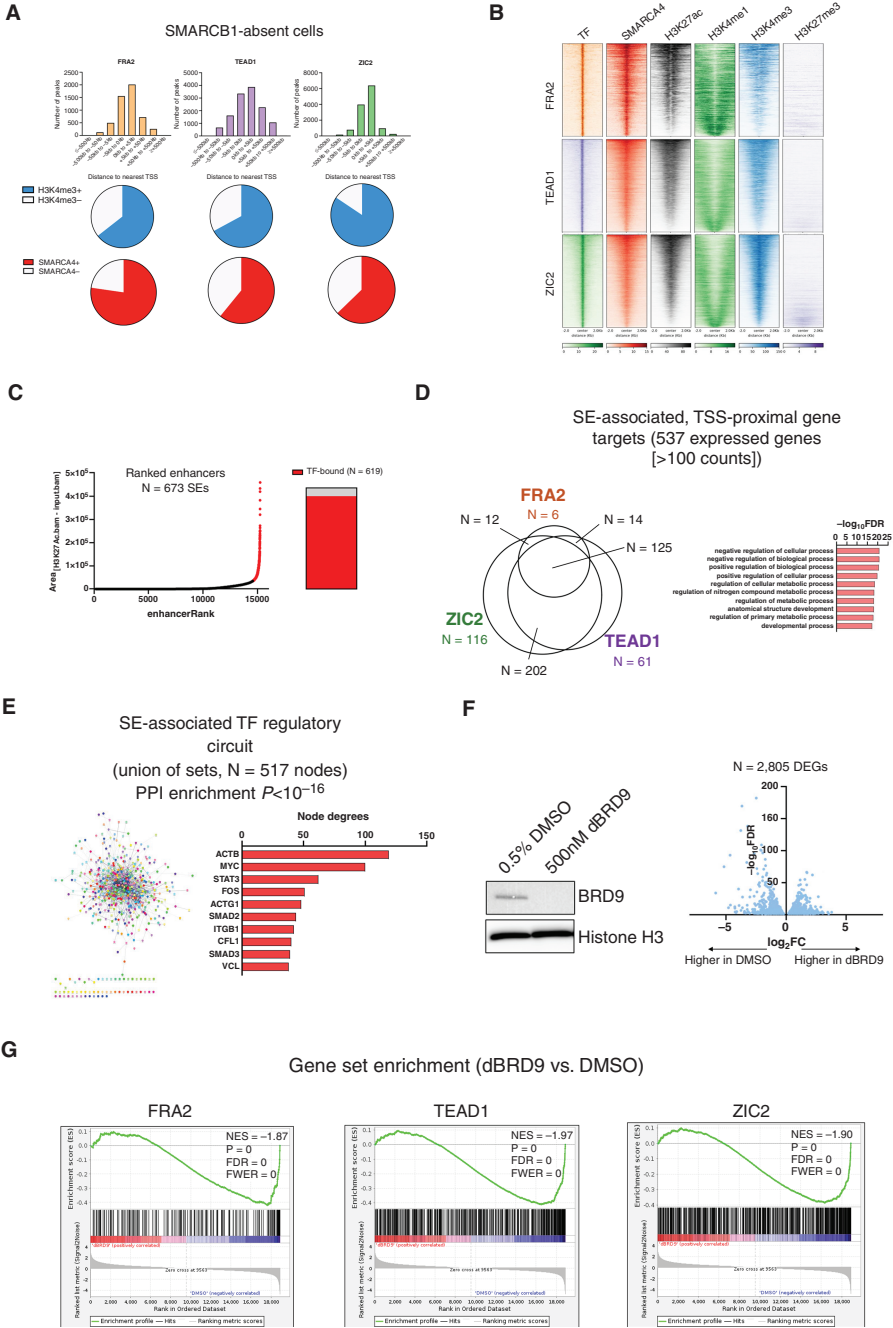
**Lineage-specific TFs are sequestered to ncBAF-dependent promoters in the absence of SMARCB1.** (**A**) Genomic distribution of TF binding sites in SMARCB1-absent cells. Relative distribution of TF peaks relative to TSSs (top) and proportion of TF peaks overlapping with H3K4me3 and SMARCA4 peaks (bottom) in SMARCB1-absent cells, highlighting promoter-associated TF occupancy. (**B**) TF binding at SMARCA4-associated promoters. ChIP-Seq profiles of H3K4me3+ and SMARCA4+ loci co-bound by each TF in SMARCB1-absent cells, demonstrating promoter binding. (**C**) ncBAF-associated super-enhancers in SMARCB1-absent cells. Ranked enhancers (ROSE) of SMARCB1-absent. Shaded call-outs highlight ncBAF-associated SEs overlapping with TF-bound active promoters (ie, ±2 kb to nearest annotated TSS, H3K4me3+, SMARCA4+). (**D**) Functional consequences of ncBAF-TF interactions. Overlap of expressed genes associated with ncBAF- and TF-bound peaks identified in C, followed by GO enrichment analysis to identify biological pathways regulated by these interactions. (**E**) Regulatory network of ncBAF-associated TF-bound genes. Schematic of regulatory circuitry showing protein-coding genes identified in (D) with the most highly integrated nodes shown. (**F**) Disrupting ncBAF alters TF-dependent transcription. BRD9 expression by Western blot (left) and volcano plot of DEGs by RNA-Seq (FDR < 0.05, right) of ATRT cells treated with either DMSO or dBRD9, illustrating the transcriptional impact of ncBAF depletion. (**G**) Gene set enrichment analysis following ncBAF inhibition. GSEA of dBRD9-treated cells, showing depletion of TF targets defined in (D) following dBRD9 treatment. BT12 data are shown.

The findings above demonstrate that SMARCB1 recruits AP-1, TEAD, and ZIC TFs to active enhancers. Given the persistence of residual SWI/SNF subunits in the absence of SMARCB1 (including non-canonical SWI/SNF, or ncBAF),^47,48^ we next sought to determine whether this relationship extends to other SWI/SNF modules. To address this possibility, we assessed the epigenomic associations of FRA2, TEAD1, and ZIC2 in SMARCB1-absent ATRT cells. In contrast to SMARCB1-restored cells, these TFs were largely sequestered to SMARCA4-associated active promoters (Figure 5A,B; Supplementary Figure S10A,B).

Relative to other SWI/SNF isotypes, ncBAF is more closely associated with promoter-proximal loci and is predicted to facilitate SE activity that is necessary for SMARCB1-deficient cell function.^47,48^ Moreover, unlike typical enhancers, BRD9-associated SEs frequently contain transcriptional start sites (TSSs) of target genes.^47^ Following our observation that SMARCB1-dependent SWI/SNF is necessary for promoter-distal TF function, we thus hypothesized that in the absence of SMARCB1, lineage-specific TFs are sequestered to promoter-proximal loci controlled by residual SE activity. To test this hypothesis, we called SEs in SMARCB1-absent cells and indeed found the majority of SWI/SNF-associated SEs contained targeted TF-bound promoters (Figure 5C; Supplementary Figure S10C). Ontological analysis of target genes demonstrated enrichment for core metabolic functions and development with a high degree of overlap between target genes (Figure 5D; Supplementary Figure S10D). Integration of gene targets into a regulatory circuit again revealed a substantial amount of inter-node cooperativity (PPI *P* < 10^−16^) with highly integrated nodes notably including MYC, STAT3, FOS, and SMAD in BT12 (Figure 5E) and MYC and CCND1 in MAF737a cells (Supplementary Figure S10E). To further demonstrate TF dependence on SWI/SNF SE activity, we treated ATRT cells with the proteolysis targeting chimera (PROTAC) degrader of BRD9 (dBRD9, targeting an ncBAF-specific subunit) and studied transcriptomic effects following BRD9 depletion (Figure 5F; Supplementary Figure S10F). BRD9 degradation significantly reduced enrichment of FRA2, TEAD1, and ZIC2 transcriptional targets except in the largest dataset, MAF737a ZIC2 (Figure 5G; Supplementary Figure S10G). These results demonstrate the universal dependence of lineage-specific TFs on SWI/SNF for execution of both promoter-proximal and -distal transcriptional programs.

### Targeting of TF Activity in SMARCB1-Absent ATRT Using Small-Molecule Inhibitors

The sequestration of lineage-specific TFs to promoters of genes necessary for cell survival led us to hypothesize that targeting TF activity might represent a therapeutic strategy for ATRT. Given the well-known challenges associated with direct pharmacological targeting of TFs and the functional redundancy of the TF families under study, we opted to target TF activity indirectly using drugs currently in human use as a proof-of-concept for this approach. To test our hypothesis, we used trametinib, a mitogen-activated protein kinase kinase (MEK) inhibitor currently in use for the treatment of select pediatric brain tumors that blocks upstream signaling necessary for AP-1 activation in MEK-activated settings^49–51^ and verteporfin, a drug used for treatment of macular degeneration that blocks assembly of co-activators necessary for TEAD transcriptional function.^52,53^

**Figure 6.**
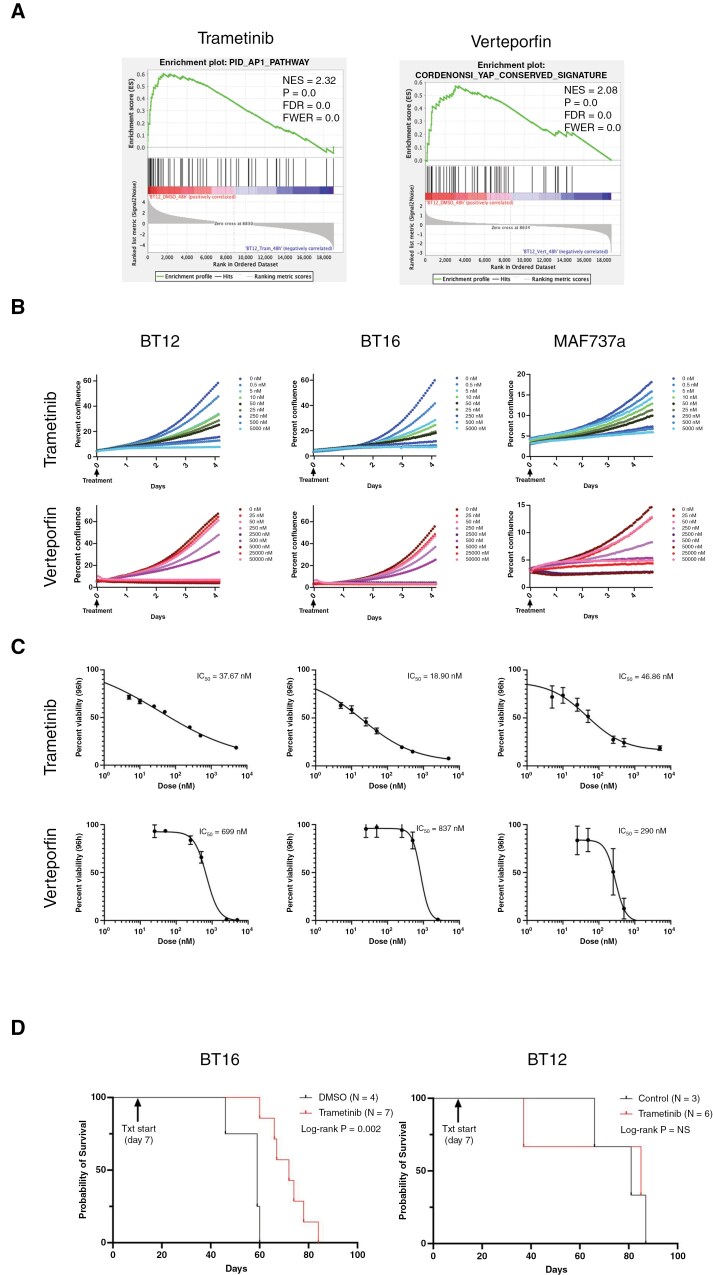
**Targeting of residual AP-1 and TEAD activity in ATRT.** (**A**) Transcriptional response to pathway inhibition. Gene set enrichment analysis of known AP-1 and YAP/TAZ/TEAD target genes in BT12 cells treated with trametinib (a MEK inhibitor that reduces downstream AP-1 activation) and verteporfin (a disruptor of YAP/TAZ-TEAD association), demonstrating pathway-specific transcriptional repression. (**B**) Cellular proliferation upon pathway inhibition. Incucyte proliferation assays of ATRT cells treated with trametinib or verteporfin at various doses, showing dose-dependent proliferation suppression. (**C**) Cellular responses to treatment. IC_50_ values of cells treated with 25 nM trametinib or 500 nM verteporfin for 48 hours, as determined by Cell Titer Blue assay. (**D**) Survival impact of trametinib treatment in ATRT xenografts. Kaplan-Meier analysis of patient-derived orthotopic xenograft ATRT models treated with trametinib. The Mantel-Cox log-rank test was used for statistical comparisons.

To confirm on-target effects, BT12 cells were first treated with 25 nM trametinib or 500 nM verteporfin for 48 hours, and RNA was extracted for analysis. Consistent with our hypothesis, treatment with trametinib and verteporfin downregulated known AP-1 and YAP/TAZ/TEAD transcriptional targets, respectively (Figure 6A). Phenotypic assessment of ATRT-MYC cells treated with escalating doses of drug demonstrated a robust cytostatic effect at an IC_50_ of 18-46 nM for trametinib and a cytotoxic effect at mid-nanomolar doses for verteporfin (Figure 6B,C). In light of these observations, we leveraged an orthotopic PDX model of ATRT in athymic nude mice using BT12 and BT16 cells. Animals were treated with either DMSO or trametinib (oral gavage, 0.5 mg/kg, 5 days per week) starting on day 7 following grafting, and overall survival was assessed. Although no survival benefit was observed in a small cohort of BT12 PDXs, treatment with trametinib significantly prolonged survival in the BT16 PDX model (Figure 6D).

## Discussion

Using patient-derived ATRT cell lines as a clinically relevant mechanistic model for the role of SWI/SNF in defining TF function, the data presented here demonstrate that SMARCB1-dependent SWI/SNF is necessary for defining the epigenomic regulatory roles of lineage-specific TFs. The AP-1 TF family plays a central role in this process, and loss of SMARCB1 in ATRT-MYC results in the specific loss of expression of the AP-1 subunit c-JUN, which orchestrates the transcriptional circuitry that is depleted in ATRT-MYC. The dependence of TF function on SWI/SNF persists in the absence of SMARCB1, where ncBAF facilitates SE-associated promoter-proximal TF function.

A growing body of evidence over the past decade implicates AP-1 as a possible central factor for mediating SWI/SNF complex recruitment to target gene enhancers in both developmental^30^ and oncologic settings.^54,55^ While the role of SWI/SNF in facilitating lineage-specific enhancer activation is now widely appreciated, by focusing on lineage-specific TFs, we sought to delineate ATRT-specific impacts of SMARCB1 loss on the enhancer landscape. Natural variation in predicted and experimentally observed TF binding sites is known to contribute to selection of strain-specific enhancers in murine models.^11,56,57^ However, human data in this regard are lacking, and more importantly, the roles of specific SWI/SNF isotypes in mediating this process have only recently been experimentally demonstrated.^32^ In this regard, our findings are novel in multiple ways. First, while other groups have demonstrated the necessity of lineage-specific TFs for SWI/SNF function, our findings demonstrate that SWI/SNF likewise is necessary for the epigenomic distribution and function of these TFs. In support of these observations, it was recently demonstrated in murine embryonic stem cells that SWI/SNF is capable of probing enhancer loci by leveraging a pioneering-type function that precedes but subsequently collaborates with lineage-specific TF binding.^58^ Our data provide critical, clinically relevant context to our evolving understanding of the interplay between lineage-specific TFs and SWI/SNF, which are increasingly appreciated as co-dependent mediators of the enhancer landscape.^58^ Second, we demonstrate enhancers associated with lineage-specific TFs converge on a developmental transcriptional program that is dependent on SMARCB1 and triggers a feed-forward loop that depends on SE-mediated upregulation of the AP-1 subunit c-JUN. This finding is particularly relevant in light of recently published single-cell RNA-Seq data in melanoma models demonstrating these AP-1 subunits as uniquely associated with a primitive cell state.^59^ Taken together, these results define a specific developmentally relevant context for SWI/SNF-TF cooperativity in which SWI/SNF complexes, not only TFs, are master regulators of cell identity. Finally, our data reveal that SMARCB1 loss unveils transcriptional dependencies that likely result from TF sequestration to promoters, and these dependencies can be targeted indirectly using small-molecule inhibitors and protein degraders. These results agree with other recent observations in a recent, separate study of ATRT cell lines of multiple molecular subtypes.^60^

Our study has multiple limitations. First, while patient-derived tumor cell lines offer valuable, clinically relevant context to epigenetic phenomena, this study lacks mechanistic comparisons to a “normal” developmental system. As the cell(s) of origin for ATRT, particularly MYC and TYR subtypes, are not yet known with certainty, this aspect cannot yet be readily addressed. SMARCB1-deficient neural stem cells (NSCs) senesce,^61^ and given the embryonal origin for ATRT and other rhabdoid tumors (ie, predicted to precede neuroectoderm formation^62^), inducible SMARCB1 knockdown in human NSCs^63^ lacks critical epigenomic context for conclusions regarding the roles of lineage-specific TFs. Given the growing role of single-cell sequencing technologies in inferring cell lineage associations in developmental models, these tools, along with mechanistically informed murine models, will be critical in identifying a meaningful “normal” model for ATRTs. A second limitation is the focus on ATRT-MYC as a mechanistic model for SMARCB1 loss. Given our finding of c-JUN loss as a critical event in ATRT-MYC, this study remained focused on this molecular subgroup. Given that our finding of SWI/SNF being required for TF binding at enhancer loci supports recent mechanistic findings in murine embryonic stem cells,^58^ we anticipate our observations reflect an evolutionarily conserved mechanism that is not restricted to ATRT. Furthermore, as different TFs are expected to interact with SWI/SNF in other subgroups of ATRT, it is not yet known which TFs may be impacted by SMARCB1 loss in other molecular subgroups. Finally, while our data demonstrated an overall survival benefit following trametinib treatment in 1 PDX model, this could not be validated in a second model. We hypothesize the results in the BT12 xenograft reflect the relative resistance to trametinib in this cell line (IC_50_ 38 nM vs. 19 nM) and the relatively conservative dose of trametinib used in this study compared to similar studies.^64,65^ These preliminary studies thus serve as important proof-of-concept for indirect pharmacological targeting of TF function in ATRT.

## Supplementary Material

noaf081_suppl_Supplementary_Materials

noaf081_suppl_Supplementary_Figure_S1

noaf081_suppl_Supplementary_Figure_S2

noaf081_suppl_Supplementary_Figure_S3

noaf081_suppl_Supplementary_Figure_S5

noaf081_suppl_Supplementary_Figure_S6

noaf081_suppl_Supplementary_Figure_S7

noaf081_suppl_Supplementary_Figure_S8

noaf081_suppl_Supplementary_Figure_S9

noaf081_suppl_Supplementary_Figure_S10

noaf081_suppl_Supplementary_Figure_S4
