# Supplementary material for: SWI/SNF complexes govern ontology-specific transcription factor function in MYC-subtype atypical teratoid rhabdoid tumor
Source: Neuro Oncol. 2025 Mar 23;27(9):2445–60. doi: 10.1093/neuonc/noaf081 (PMC12526124; doi:10.1093/neuonc/noaf081)
Supplement: noaf081_suppl_Supplementary_Materials [file noaf081_suppl_supplementary_materials.docx]

**Supplementary Information**

**SWI/SNF complexes govern ontology-specific transcription factor function in MYC-subtype atypical teratoid rhabdoid tumor**

Cody L. Nesvick, Liang Zhang, Yuqian Yan, Alexander Q. Wixom, Feda H. Hamdan, Jizhi Ge, Jacob B. Anderson, Alexandre Gaspar-Maia, Steven A. Johnsen, David J. Daniels

**SUPPLEMENTARY FIGURE LEGENDSD**

**Supplementary Figure 1. Assessment of inter-group variability in transcriptomic data from ATRT cell lines.** (**A**) Principal component analysis (PCA) of RNA-Seq data from SMARCB1-absent and -restored cell lines. (**B**) Venn diagram illustrating relative overlap of significantly up-regulated differentially expressed genes (DEGs) between cell lines. Gene ontology (GO) analyses for up-regulated DEGs shared between all cell lines are shown. (**C**) Venn diagram illustrating relative overlap of significantly down-regulated differentially expressed genes (DEGs) between cell lines. Gene ontology (GO) analyses for down-regulated DEGs shared between all cell lines are shown.

**Supplementary Figure 2. SMARCB1 restoration induces senescence in ATRT cell lines.** (**A**) Indicated cell lines were transduced with a lentiviral vector containing either a empty vector (red fluorescent protein, RFP) or SMARCB1 for 5 days and harvested for western blot analysis. (**B**) Indicated cell lines generated from (A) were further used for Incucyte proliferation assays. (**C and D**) Clonogenic assays were performed in select ATRT-MYC and ATRT-SHH cell lines expressing either RFP or SMARCB1. The number of colonies was quantified in (**C**) with representative images in (**D**). The P value was calculated by the unpaired two-tailed Student's t-test; **P* < 0.05, ****P* < 0.001. (**E**) Relative enrichment of E2F target genes (necessary for G1-S transition) and senescence-associated genes in SMARCB1-absent and -restored cells. Normalized enrichment scores and statistical significance values are shown.

**Supplementary Figure 3. Expression of AP-1 subunits, TEAD and ZIC family transcription factors (TFs) in SMARCB1-absent and -restored ATRT cells (related to Fig. 2). (A)** Expression of AP-1 subunits, TEAD and ZIC TFs by RNA-Seq. Expression is shown as the log2 of (transcripts per million [TPM] +1). **(B)** Western blot illustrating expression of the indicated transcription factors and subunits.

**Supplementary Figure 4. Active enhancer associations of FRA2, TEAD1 and ZIC2 in MAF737a cells.** FRA2 ChIP-Seq peak overlap and motif enrichment of the FRA2 peak set (union of peaks) in SMARCB1-absent and -restored MAF737a cells. **(B)** Read pileups of SMARCB1-dependent active enhancers associated with FRA2, TEAD1 and ZIC2. **(C)** Relative overlap of TF peaks associated with gained active enhancers following SMARCB1 restoration. (**D**) Gene set enrichment analysis (GSEA) TF-associated enhancer target gene expression assessed by RNA-Seq. Enhancer targets were predicted by proximity to known regulatory elements (GeneHancer). (**E**) Venn diagram illustrating overlap in TF-associated enhancer target genes upregulated following SMARCB1 restoration.

**Supplementary Figure 5. Epigenomic associations of the AP-1 subunit FRA2, TEAD1 and ZIC2 in SMARCB1-absent and -restored ATRT cells.** TF peaks are sorted by the indicated associations with promoters (H3K4me3), active enhancers (H3K27ac without H3K4me3) and gained enhancers (gained H3K27ac contributing to ROSE-assigned enhancer) in BT12 (A) and MAF737a (B) cells.

**Supplementary Figure 6. Gene ontology (GO) analysis of up-regulated genes targeted by SMARCB1-dependent enhancers**. GO analyses are organized by each TF associated with gained active enhancers in BT12 cells.

**Supplementary Figure 7. Epigenomic associations of TEAD1 and ZIC2 in SMARCB1-absent and -restored DJD29 cells**. **(A)** TF peaks are sorted by the indicated associations with promoters (H3K4me3), active enhancers (H3K27ac without H3K4me3) and gained enhancers (gained H3K27ac contributing to ROSE-assigned enhancer). **(B)** Detailed view of TEAD1- and ZIC2-associated gained enhancers shown in A. **(C)** Relative overlap of TF peaks associated with gained active enhancers following SMARCB1 restoration. (**D**) Gene set enrichment analysis (GSEA) TF-associated enhancer target gene expression assessed by RNA-Seq. Enhancer targets were predicted by proximity to known regulatory elements (GeneHancer). (**E**) Venn diagram illustrating overlap in TF-associated enhancer target genes upregulated following SMARCB1 restoration.

**Supplementary Figure 8. Transcription-factor mediated interactions underlie a conserved c-JUN-oriented core regulatory circuit.** (**A**) Network cooperativity analysis using annotated protein-protein interactions and up-regulated target genes of enhancers associated with FRA2, TEAD1 and ZIC2 ChIP-Seq peaks as input. The union of networks, a shared regulatory circuit between TF-associated enhancers is at right with the most highly integrated network nodes shown. (**B**) Single-nearest neighbors of c-JUN forming the c-JUN core regulatory circuit (CRC). (**C**) Venn diagram demonstrating shared members of the c-JUN CRC between BT12 qnd MAF737a cell lines. (**D**) *JUN* mRNA and c-JUN protein expression by RNA-Seq and Western blot, respectively. (**E**) Illustration of *JUN-*associated SE in MAF737a. The P value was calculated using unpaired two-tailed Student's t-test; ****P* < 0.001.

**Supplementary Figure 9. Over-expression of c-JUN is not sufficient to reproduce phenotypic changes and enhancer reprogramming in the absence of SMARCB1 (related to Fig. 4).** (**A and B**) BT12 and MAF737a were overexpressed with cJUN and harvested for Western blot of cJUN (**A**) and Incucyte proliferation assays (**B**). (**C**) H3K27ac and H3K4me1 ChIP-Seq read pileups at loci of SMARCB1-dependent, AP-1-associated enhancers in BT12 and MAF7373a cell lines.

**Supplementary Figure 10. Lineage-specific TFs are sequestered to ncBAF-dependent, SE-enhanced promoters in the absence of SMARCB1 (MAF737a).** (**A**) Genomic distribution of FRA2, TEAD1 and ZIC2 in SMARCB1-absent MAF737a cells. (**B**) Read pileups demonstrating H3K4me3+, BRG1+ loci co-bound by each TF in SMARCB1-absent cells. (**C**) Ranked enhancers (ROSE) and fraction of ncBAF-associated SEs overlapping with TF-bound active promoters (± 2kb to nearest annotated TSS, H3K4me3+, BRG1+). (**D**) Overlap of expressed genes associated with ncBAF-, TF-bound peaks identified in C with predicted GO enrichment analysis. (**E**) Predicted regulatory circuit comprised of protein-coding genes identified in C. (**F**) BRD9 expression by Western blot and volcano plot of DEGs (FDR < 0.05) following dBRD9 treatment for 72h. (**G**) Gene set enrichment analysis of genes identified in C stratified by TF family.

**Supplementary Methods**

**Cell culture**

BT12 and BT16 cell lines were provided by the Childhood Cancer Repository, courtesy of Dr. Peter Houghton. MAF737a cells were provided by Dr. Jean Mulcahy Levy of the University of Colorado. BT37 was provided by Dr. Eric Raabe of Johns Hopkins University. DJD029 was derived from a previously untreated patient at Mayo Clinic Rochester. CHLA02, CHLA04 and CHLA05 were obtained from the laboratory of Dr. James Rutka (Toronto Hospital for Sick Children). Cell-line specific *SMARCB1* alterations were confirmed by Sanger sequencing in BT12 and BT16 ^34^, and cell line identity was confirmed for BT12 and MAF737a using short tandem repeat (STR) analysis. Molecular subgrouping analysis for all cell lines except for DJD29 was performed using a clinical-grade RNA-Seq-based nanostring assay (Hospital for Sick Children). DJD29 was classified using bisulfite methylation sequencing (National Institutes of Health) and the Heidelberg Brain Tumor Classifier (version 12).^7^ Cell culture conditions are provided in Supplementary Table 1.

Vectors for *SMARCB1, JUN,* and *RFP* controls were obtained from Horizon Discovery (Supplementary Table 2) and packaged in lentiviral particles for efficient transduction. *SMARCB1* variants were synthesized by GenScript into a pGenLenti backbone (custom synthesis). HEK293T cells were used for viral production, according to the Broad Institute RNAi Consortium (TRC) guidelines (https://portals.broadinstitute.org/gpp/public/dir/download?dirpath=protocols/production&filename=TRC%20shRNA%20sgRNA%20ORF%20Low%20Throughput%20Viral%20Production%20201506.pdf). Following transduction, cells were selected in the appropriate antibiotic(s) for 5 days prior to being used for further assay. SMARCB1-restored ATRT cells were not further passaged following transduction and selection.

For studies utilizing molecular therapeutics, cells were treated with the indicated drugs and doses in 0.5% DMSO (TargetMol) or 0.5% DMSO alone for 48 hours (trametinib, verteporfin) or 72 hours (dBRD9) prior to harvesting. Complete or near-complete loss of BRD9 expression was confirmed by Western blot prior to additional assays.

**Western blot**

Cells were lysed using RIPA lysis buffer with cOmplete mini protease inhibitor cocktail (Roche), and protein concentration was measured using the Thermo BCA kit. Ten micrograms of protein were loaded per blot. Protein samples were run on 12.5% SDS-PAGE gels and transferred to a PVDF membrane overnight (all reagents from Bio-Rad). Membranes were incubated in primary antibody overnight at 4°C, rinsed thoroughly in PBS-T and incubated in species-specific antibody for two hours at 4°C. SuperSignal West Pico (Thermo) was used for HRP-conjugated secondary antibody detection. Antibodies used in this study are listed in Supplementary Table 3.

**RNA extraction, library preparation and sequencing**

Cell pellets were rinsed once in cool PBS and lysed in Qiazol (Qiagen). RNA was extracted using a microRNEasy kit (Qiagen). Further processing of extracted RNA was performed by Novogene (Beijing, China). Briefly, RNA amount and purity was confirmed using a nanophotometer (Implen) and Agilent 2100 Bioanalyzer. One microgram of pure RNA was barcoded and amplified using the NEBNext UltraTM RNA Library Prep Kit for Illumina Sequencers (New England Biolabs). cDNA libraries were then size-selected to an optimal length of 150 – 200bp using AMPure XP magnetic beads (Beckman Coulter) and pooled for sequencing. cDNA sequencing was performed using an Illumina Novaseq 6000 according to manufacturer specifications.

**ChIP-Seq**

Cells were fixed in suspension in 1% formaldehyde for 20 minutes, and fixation was quenched with 125 μM glycine for five minutes. Fixates were centrifuged, rinsed twice in cold PBS, and nuclei were extracted with Nelson nuclear extraction buffer (150 mM NaCl, 20 mM EDTA pH 8, 50 mM Tris HCl pH 7.5, 0.5% NP40, 1% Triton X-100, 20 mM NaF in MBWG). Nuclear pellets were then resuspended in Gomes nuclear lysis buffer (150 mM NaCl, 20 mM EDTA pH 8, 50 mM Tris HCl pH 8, 1% NP40, 20 mM NaF, 0.5% sodium deoxycholate, 0.1% freshly added SDS in MBGW) to an estimated final concentration of 10^6^ nuclei / 100 μL. Chromatin slurry was then sonicated using a Diagenode Bioruptor Pico to a goal mean fragment size of 100 – 500 bp, and insoluble debris were removed by centrifugation. Chromatin extracts were pre-cleared for one hour prior to immunoprecipitation.

Antibody was added to pre-cleared chromatin extracts and incubated overnight at 4°C with rotation. Antibody concentrations used for ChIP-Seq are presented in Supplementary Table 3. ChIP-grade magnetic protein A/G beads (Thermo) were added to lysates and incubated on a wheel at 4°C for two hours. Antibody-bead complexes were pelleted using a DynaMag-2 magnet (Invitrogen) and serially washed with Gomes lysis buffer (x1), Gomes wash buffer (20mM EDTA pH 8, 100mM Tris HCl pH 8.5, 1% NP40, 20mM NaF, 1% sodium deoxycholate, 500mM freshly added LiCl). Washed complexes were then incubated for 30 minutes in RNAse A (Thermo) at 37°C, 800RPM and then overnight in proteinase K (Thermo) at 65°C, 800RPM for de-crosslinking. DNA was then extracted using phenol-chloroform and precipitated with ethanol.

The Diagenode Microplex v2 or v3 kit was used for library preparation with equal starting amounts of precipitated DNA between conditions for each target under study. Library size selection was performed using sparQ PureMag beads (Quantabio) with a goal fragment size of 200 – 500 bp, which was confirmed using a Bioanalyzer 2100 (Agilent). Pooled libraries were sequenced on an Illumina HiSeq 4000.

**Supplementary Table 1. Media composition for patient-derived cell lines.**

| Cell lines | Media name | Media contents |
| --- | --- | --- |
| BT12, BT16, MAF737a | NA | RPMI1640 with glutaMAX (Fisher Scientific) supplemented with 10% FBS (Atlanta Biologicals) |
| DJD29 | Tumor stem cell medium (TSM) | Neurobasal supplement (without vitamin A) (Life Technologies), supplement B27 (without vitamin A) (Life Technologies), 4 µg/mL heparin (Sigma-Aldrich), 20 ng/mL human EGF (PeproTech), 20 ng/mL human b-FGF (PeproTech), 20 ng/mL human PDGF-AA, 20 ng/mL human PDGF BB (Shenandoah Biotechnology). |
| CHLA02, CHLA04, CHLA05 | NA | DMEM/F12 (Life Technologies), supplement B27 (without vitamin A) (Life Technologies), 20 ng/mL human EGF (PeproTech), 20 ng/mL human b-FGF (PeproTech). |

**Supplementary Table 2. Expression constructs.**

| **Transgene** | **Selection cassette** | **Supplier** | **Catalog No.** |
| --- | --- | --- | --- |
| *RFP* (negative control) | Blasticidin | Horizon Discovery | OHS5832 |
| *SMARCB1* | Blasticidin | Horizon Discovery | OHS5879-202617080 |
| *JUN* | Blasticidin | Horizon Discovery | OHS5898-202622419 |

**Supplementary Table 3. Antibodies.**

| **Target Name** | **Supplier** | **Species** | **Catalog No.** | **Dilution** |
| --- | --- | --- | --- | --- |
| BAF47 / SMARCB1 | Cell Signaling | Rabbit | D8M1X | 1:10,000 (WB) |
| BRD9 | Cell Signaling | Rabbit | E9R2I | 1:1,000 (WB) |
| BRG1 / SMARCA4 | Abcam | Rabbit | ab110641 | 12 ug (ChIP); 1:2000 (WB) |
| FOS | Cell Signaling | Rabbit | 9F6 | 1:1,000 (WB) |
| FOSB | Cell Signaling | Rabbit | 5G4 | 1:1,000 (WB) |
| FRA1 | Cell Signaling | Rabbit | D80B4 | 1:1,000 (WB) |
| FRA2 | Cell Signaling | Rabbit | D2F1E | 1:50 (ChIP); 1:1,000 (WB) |
| H3K4me1 | Diagenode | Rabbit | C15410194 | 2 ug (ChIP) |
| H3K4me3 | Diagenode | Rabbit | C15410003 | 2 ug (ChIP) |
| H3K27ac | Diagenode | Rabbit | C15410196 | 2 ug (ChIP) |
| H3K27me3 | Diagenode | Rabbit | C15410195 | 2 ug (ChIP) |
| Histone H3 | Cell Signaling | Mouse | 96C10 | 1:1,000 (WB) |
| c-JUN | Cell Signaling | Rabbit | 60A8 | 1:1,000 (WB) |
| JUNB | Cell Signaling | Rabbit | C37F9 | 1:1,000 (WB) |
| JUND | Cell Signaling | Rabbit | D17G2 | 1:1,000 (WB) |
| TEAD1 | ActiveMotif | Rabbit | 61643 | 1:50 (ChIP); 1:2,000 (WB) |
| ZIC2 | Abcam | Rabbit | ab150404 | 12.7 ug (ChIP); 1:2,000 (WB) |
