# Supplementary figures and images for: SWI/SNF complexes govern ontology-specific transcription factor function in MYC-subtype atypical teratoid rhabdoid tumor
Source: Neuro Oncol. 2025 Mar 23;27(9):2445–60. doi: 10.1093/neuonc/noaf081 (PMC12526124; doi:10.1093/neuonc/noaf081)

A

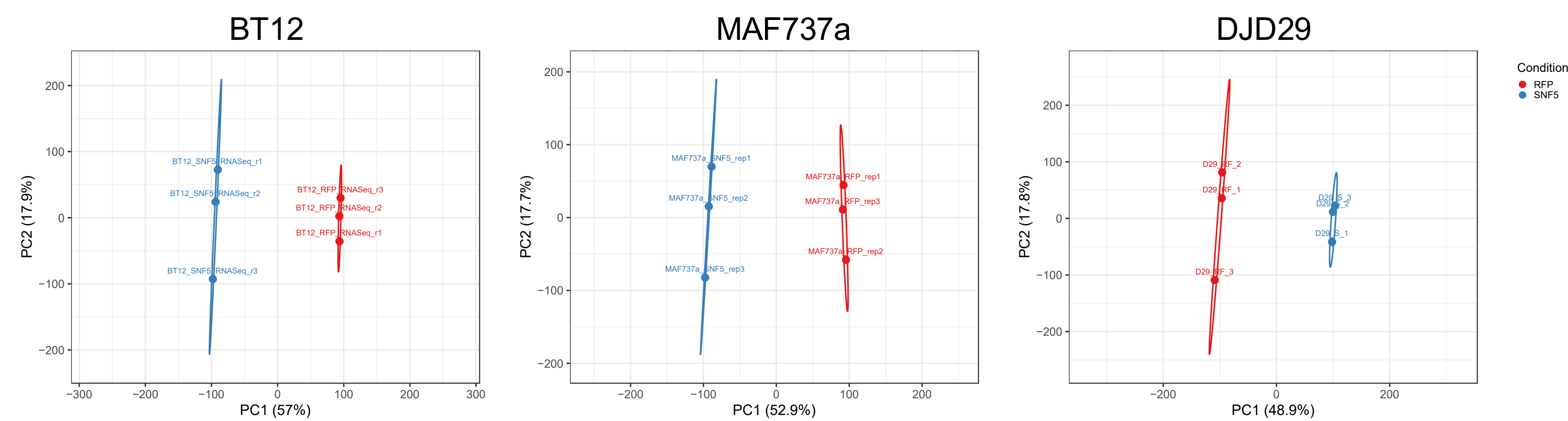

B

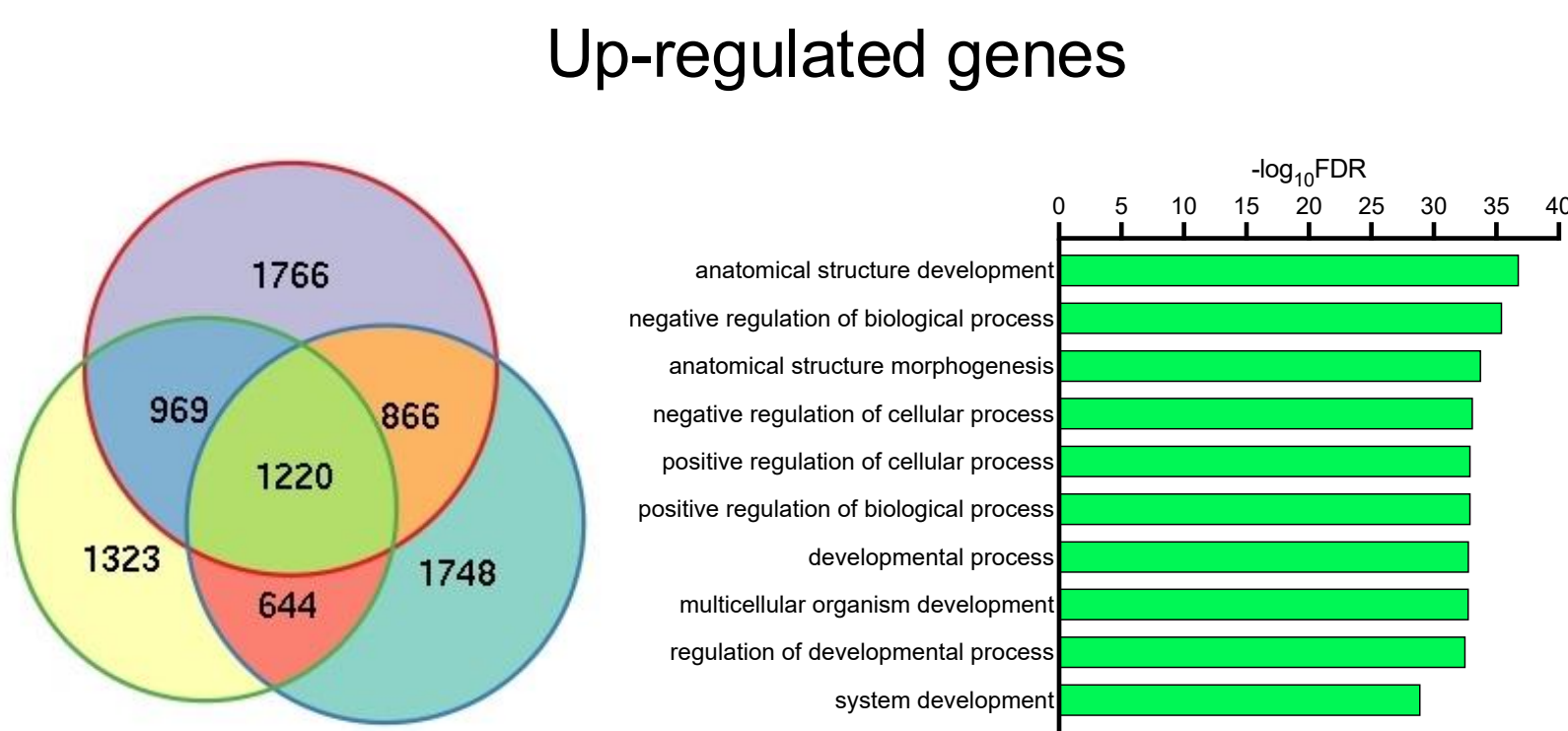

C

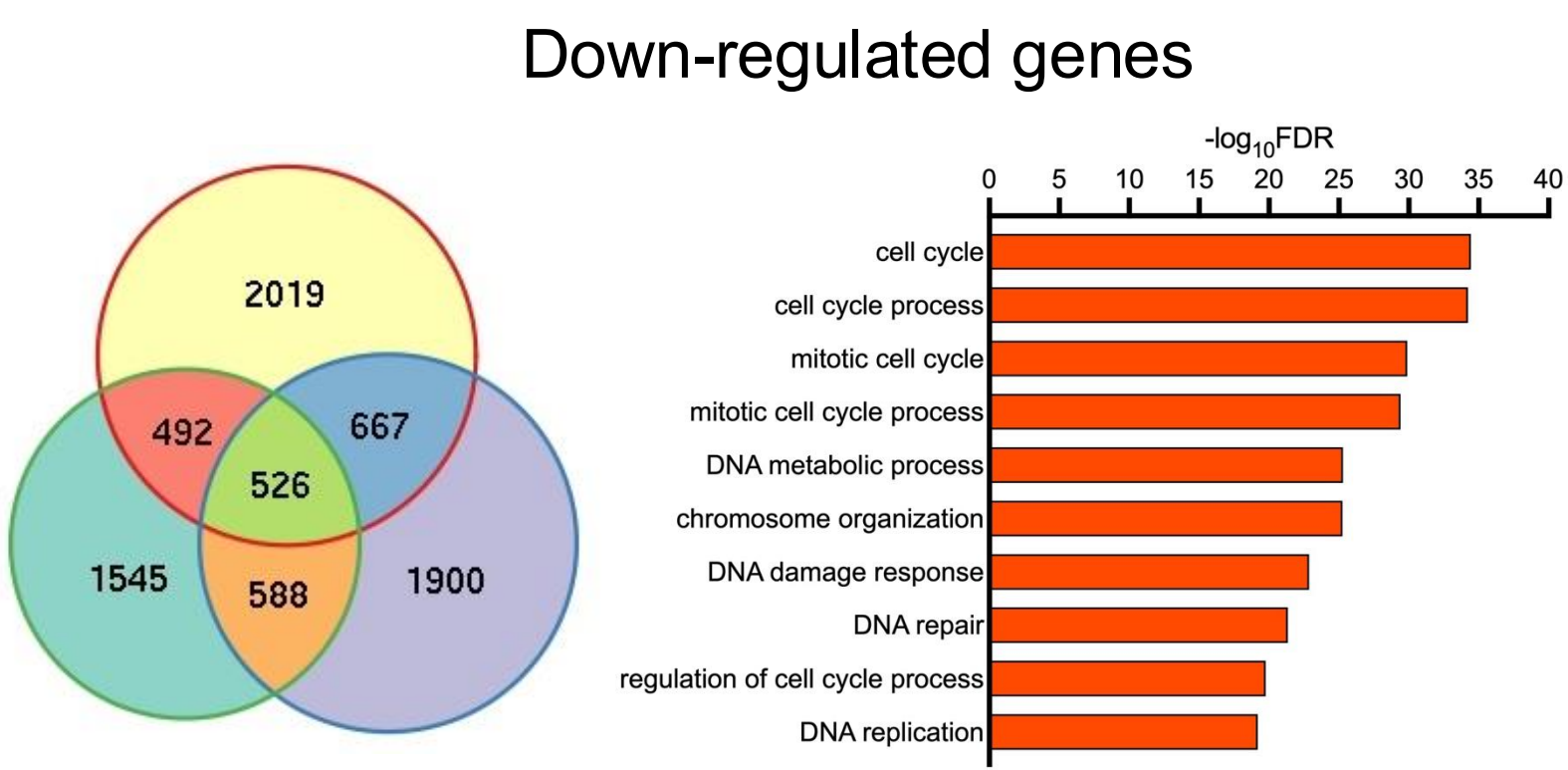

Supplement: noaf081_suppl_Supplementary_Figure_S1 [file noaf081_suppl_supplementary_figure_s1.pdf]

**A**

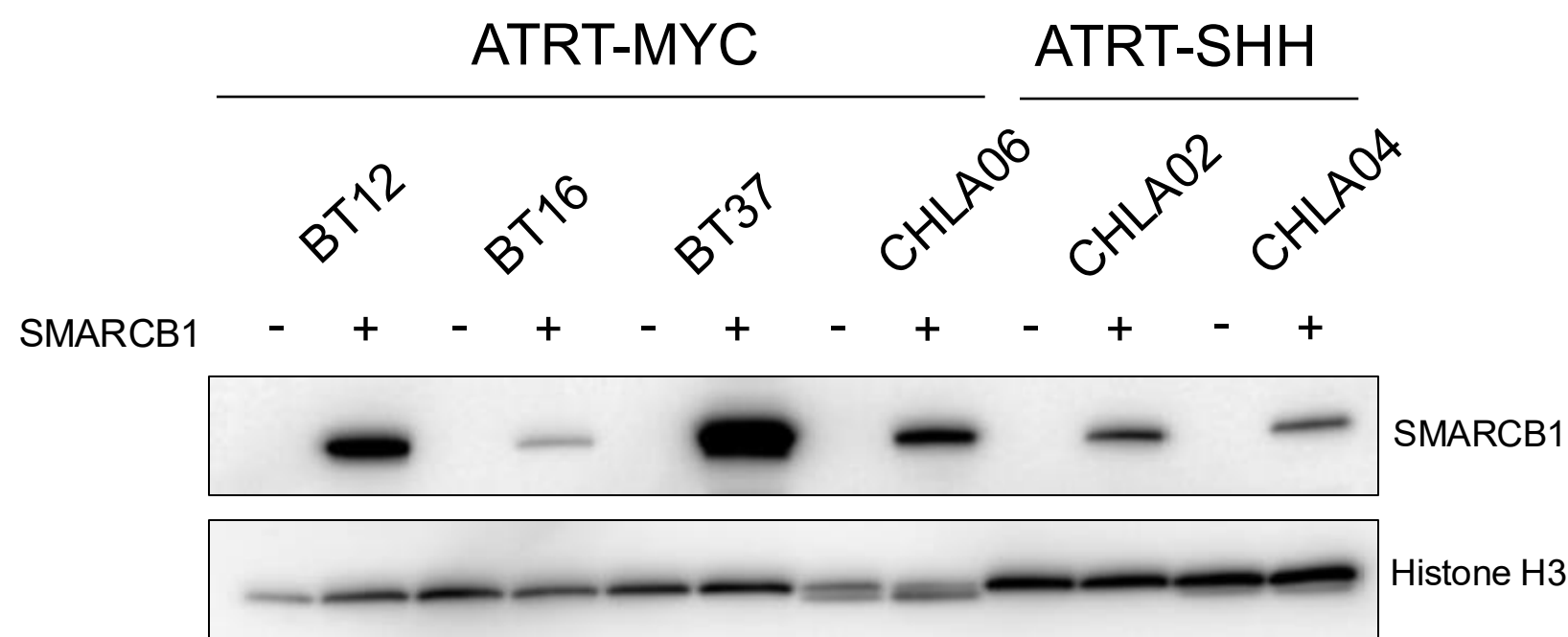

**B**

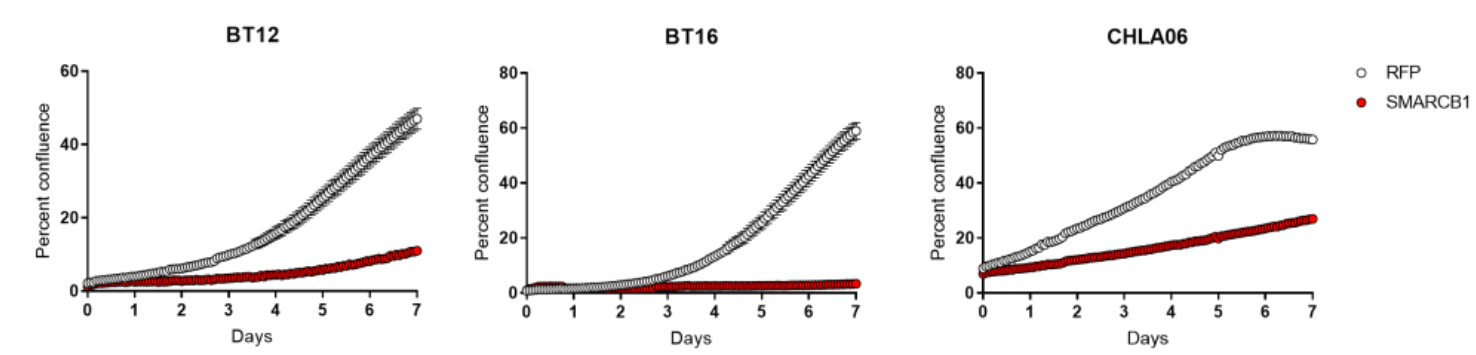

**D**

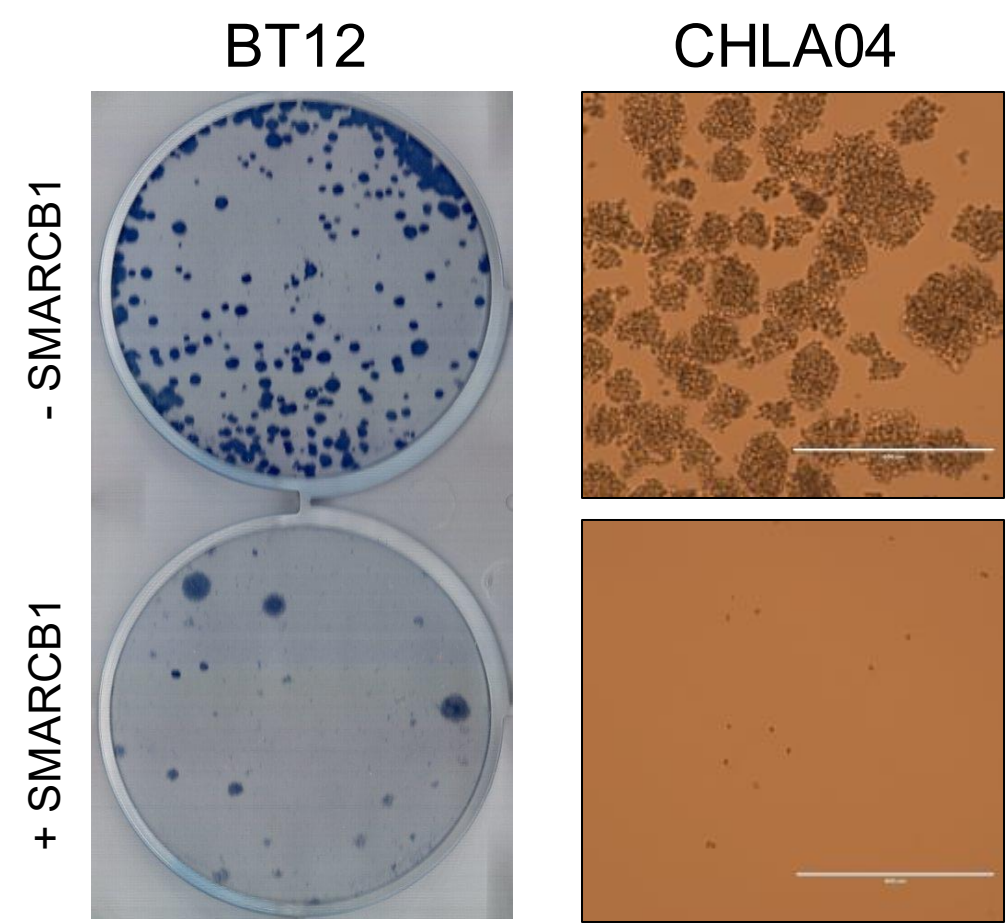

**C**

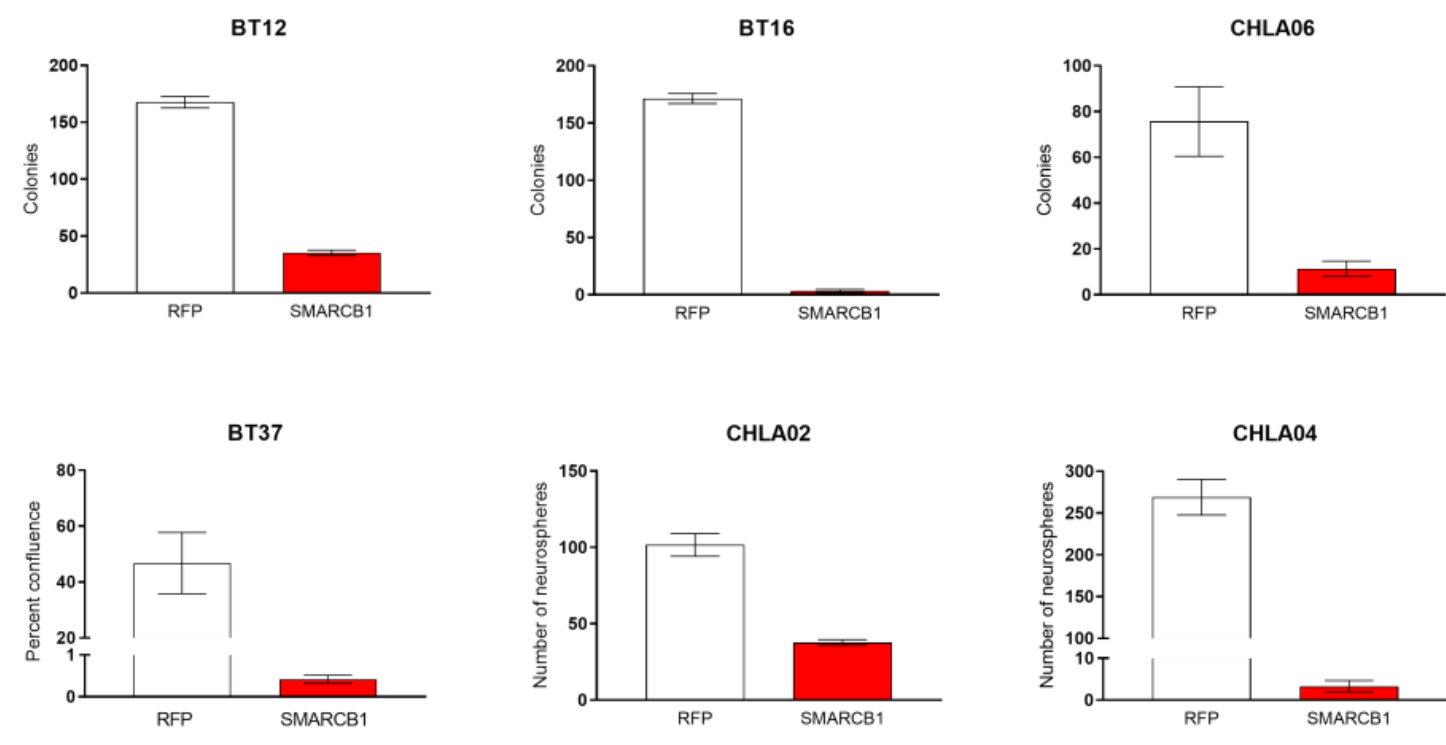

**E**

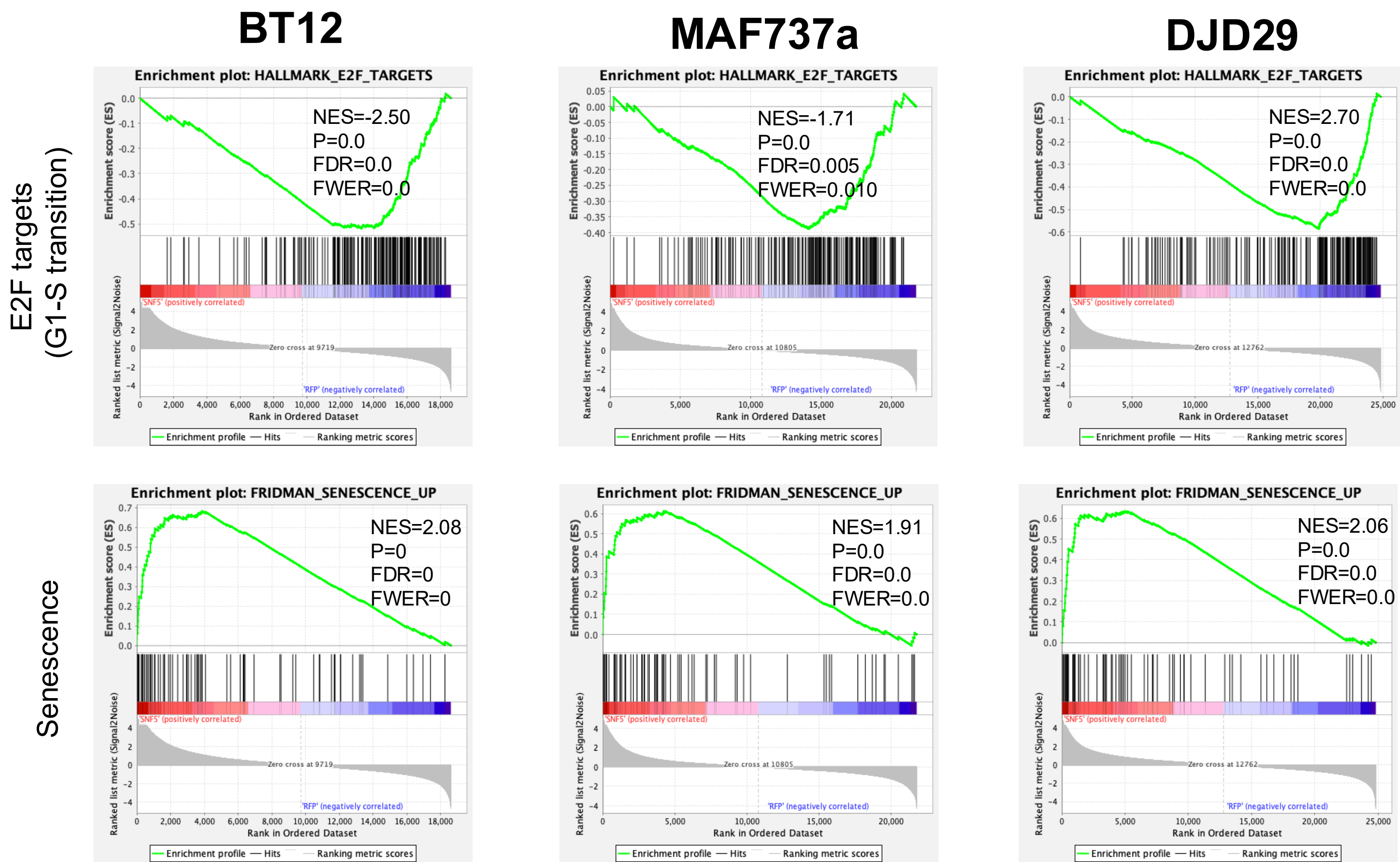

Supplement: noaf081_suppl_Supplementary_Figure_S2 [file noaf081_suppl_supplementary_figure_s2.pdf]

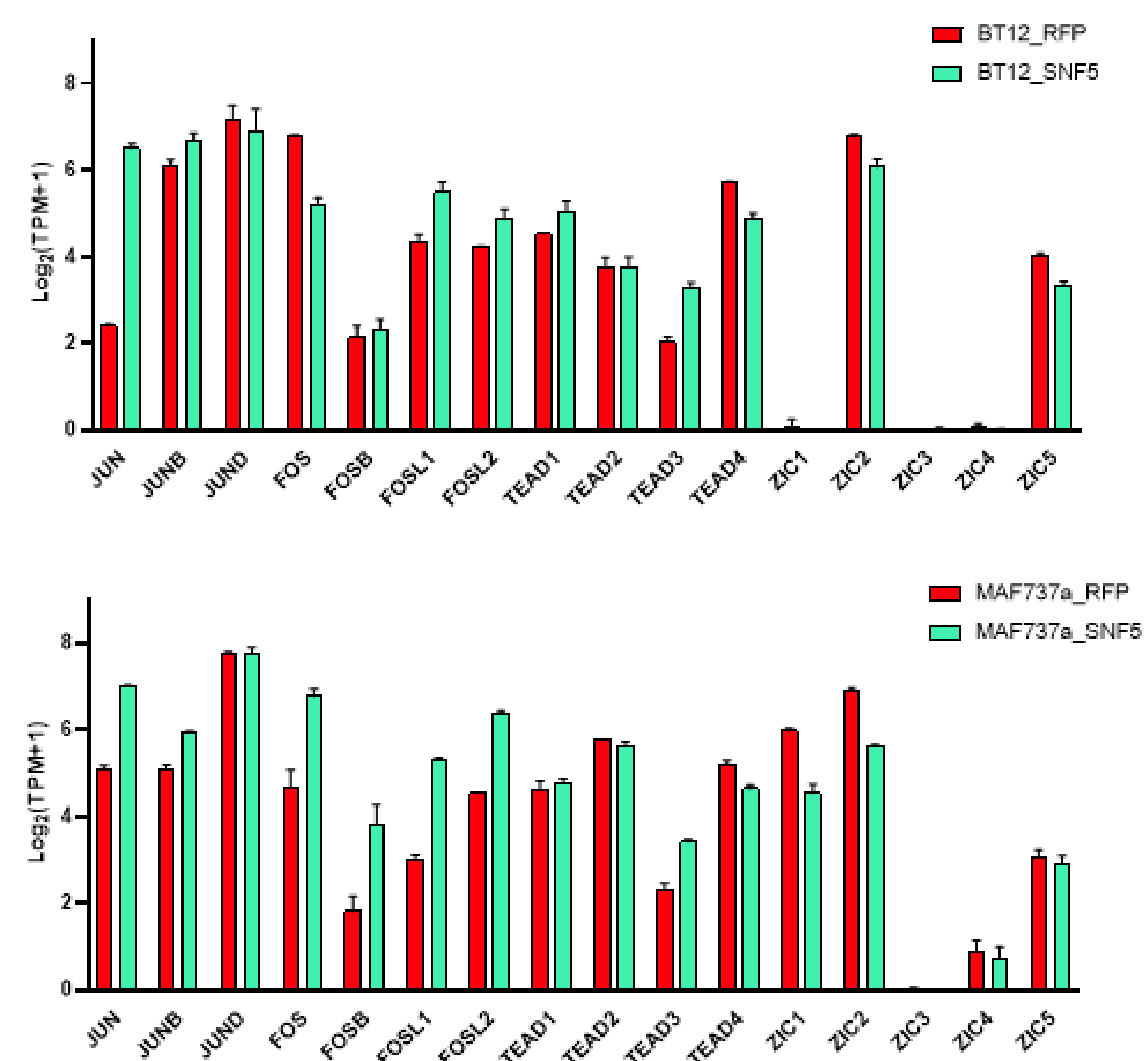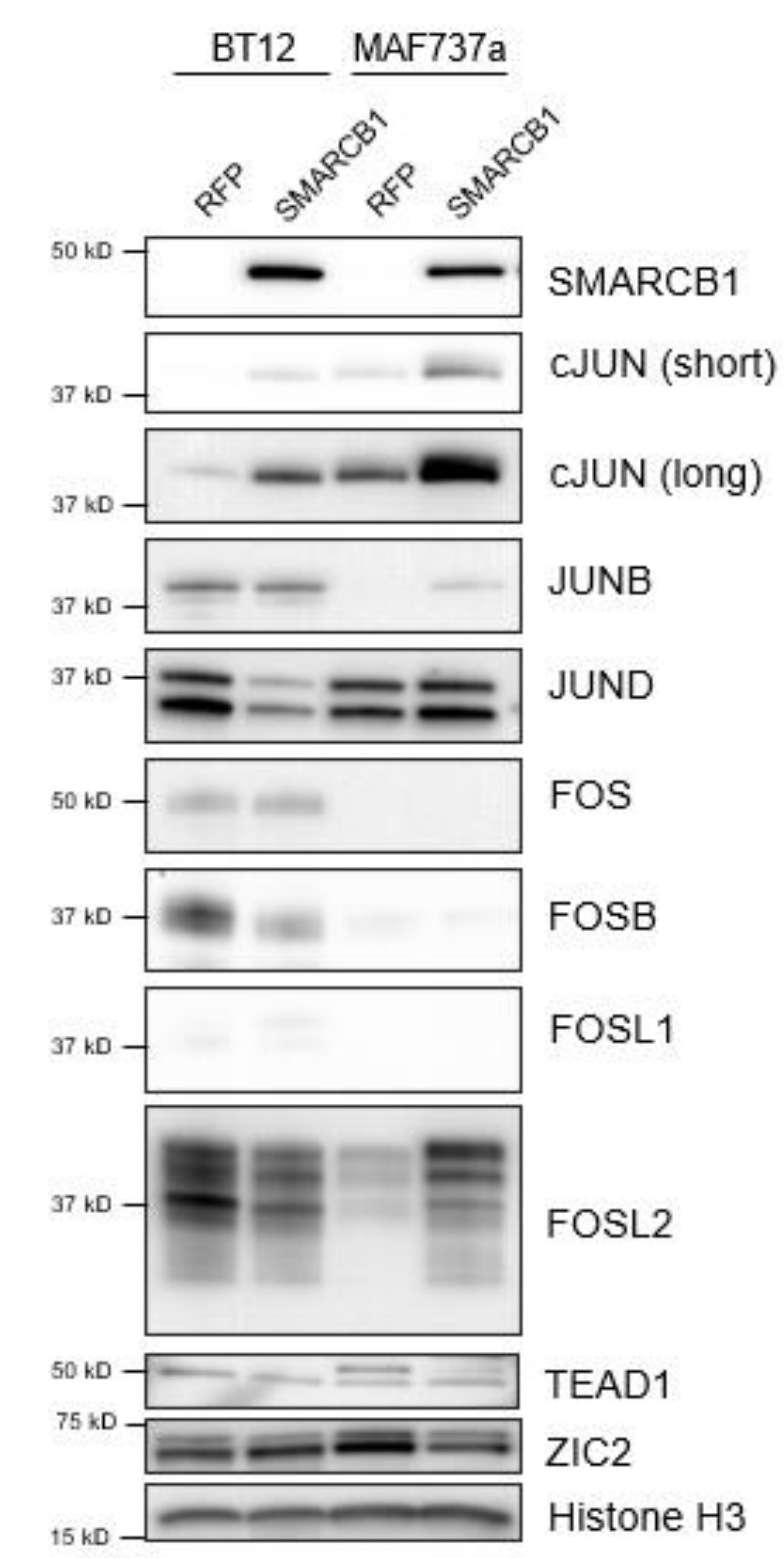

Supplement: noaf081_suppl_Supplementary_Figure_S3 [file noaf081_suppl_supplementary_figure_s3.pdf]

Epigenomic Associations of Transcription Factors

A

BT12

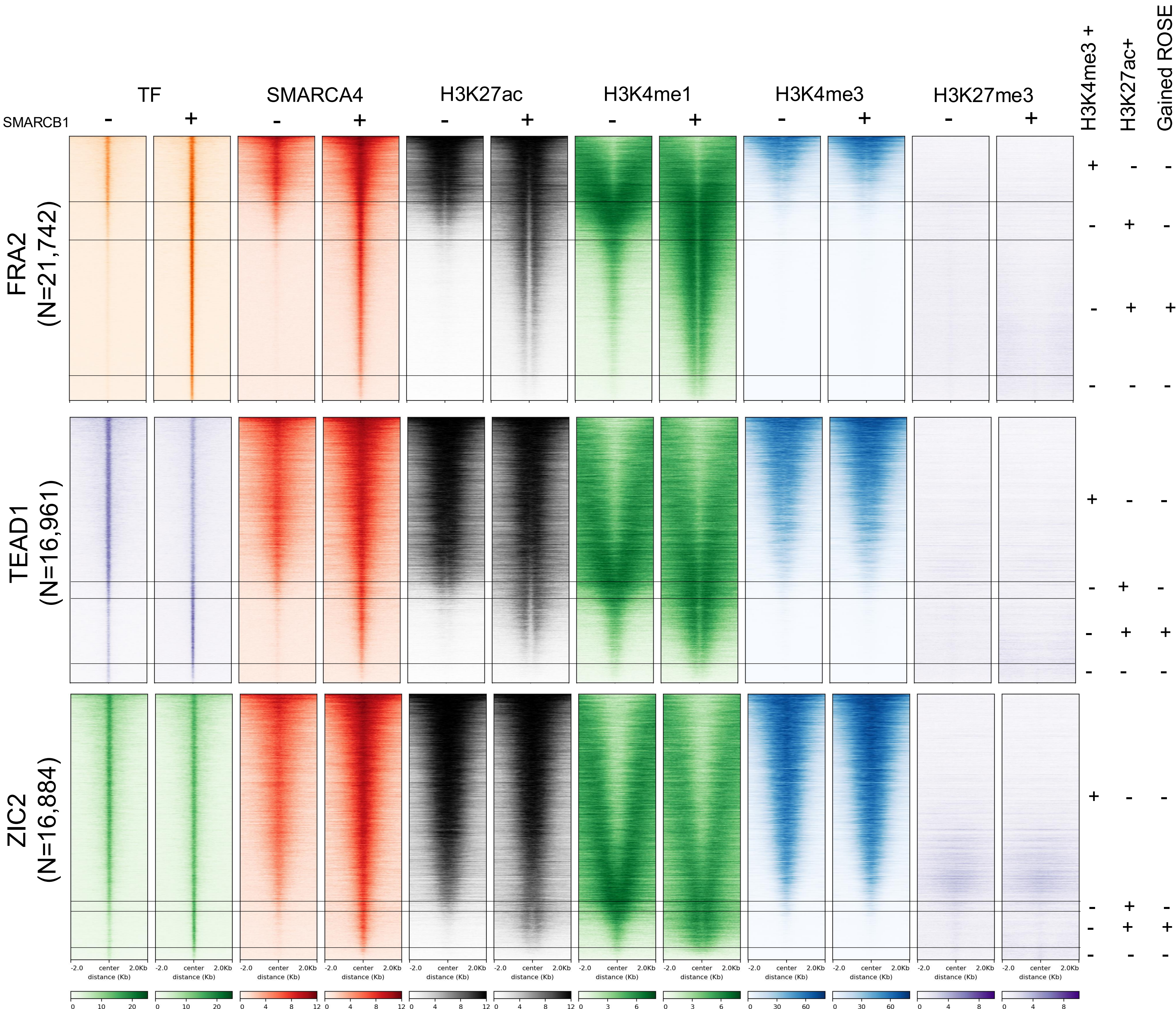

B

MAF737a

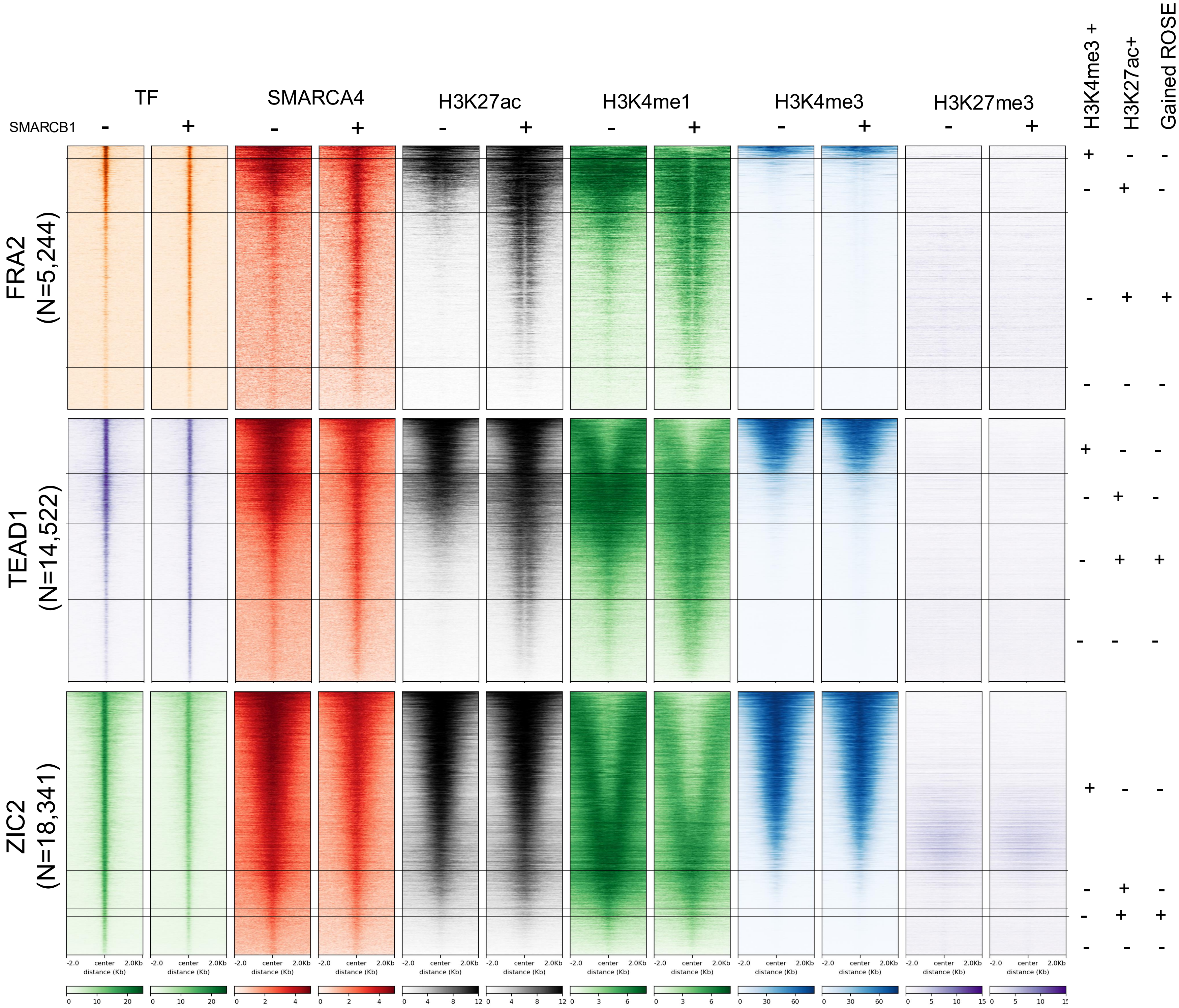

Supplement: noaf081_suppl_Supplementary_Figure_S5 [file noaf081_suppl_supplementary_figure_s5.pdf]

FRA2

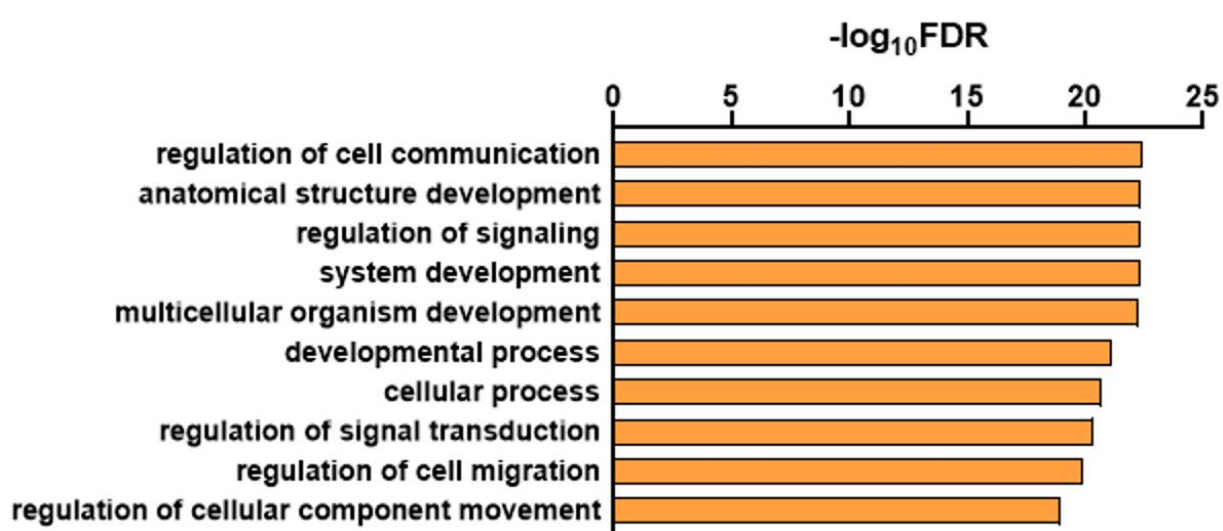

TEAD1

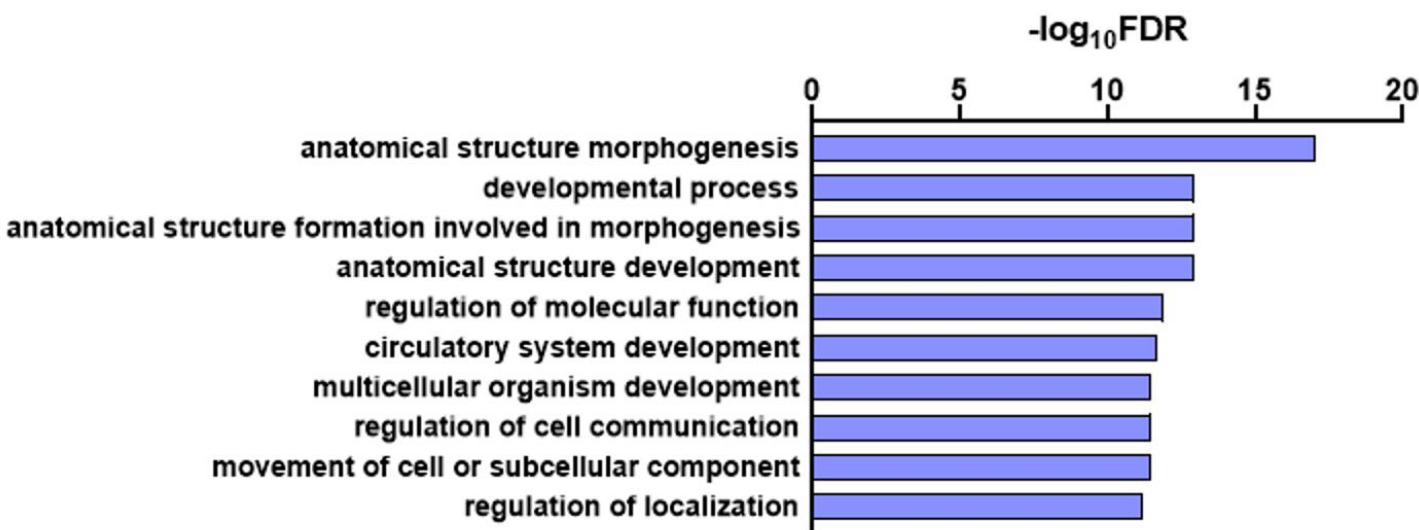

ZIC2

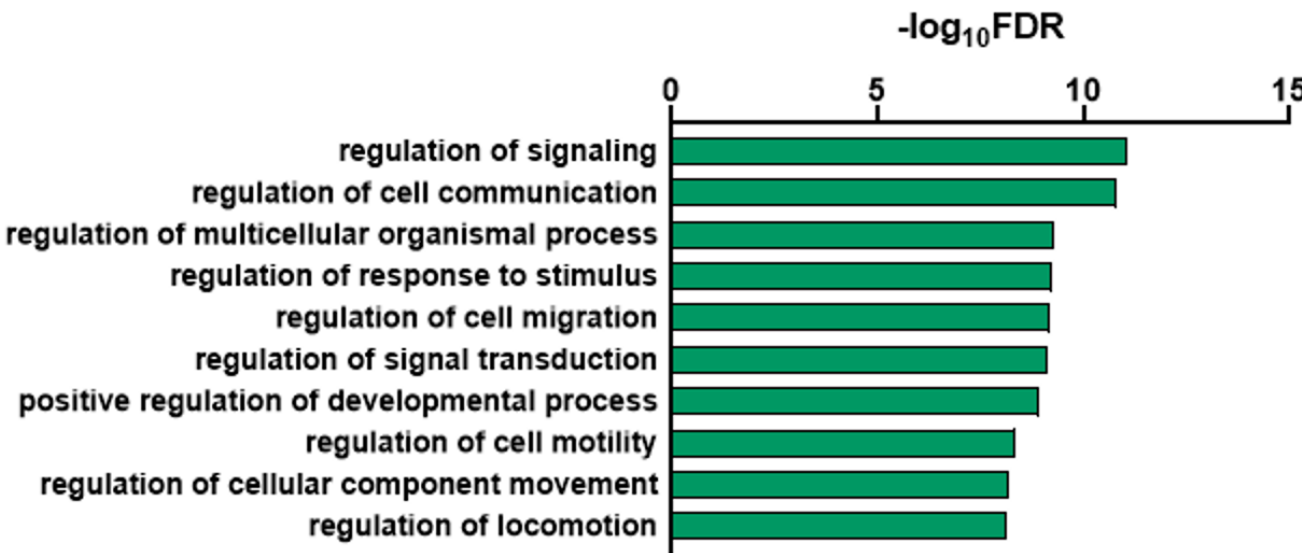

Supplement: noaf081_suppl_Supplementary_Figure_S6 [file noaf081_suppl_supplementary_figure_s6.pdf]

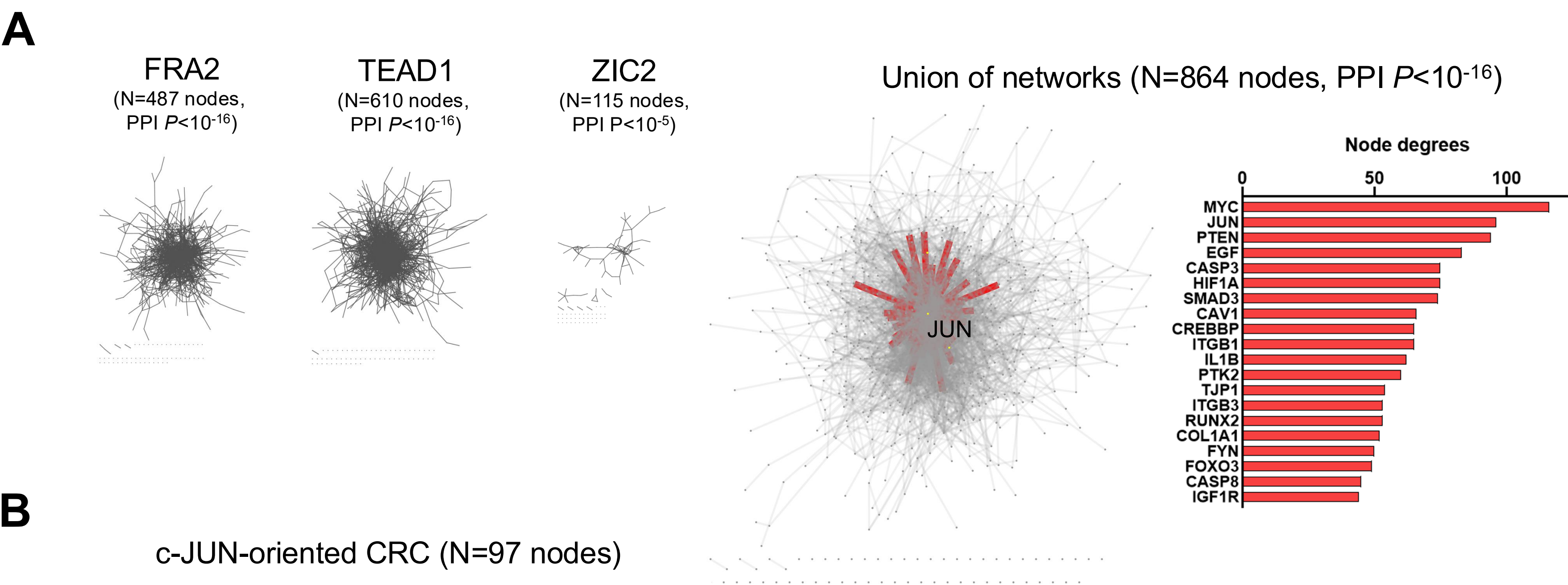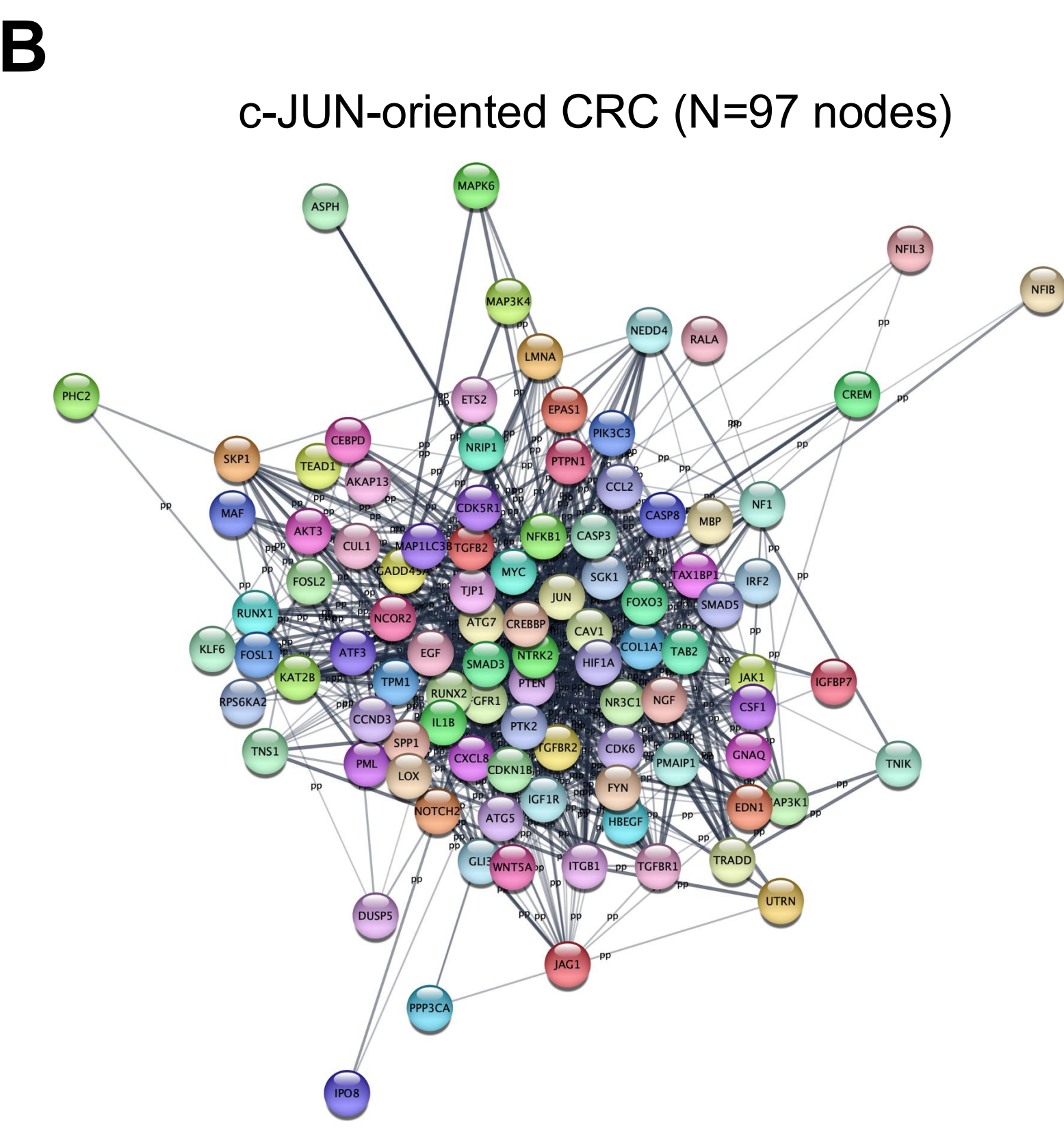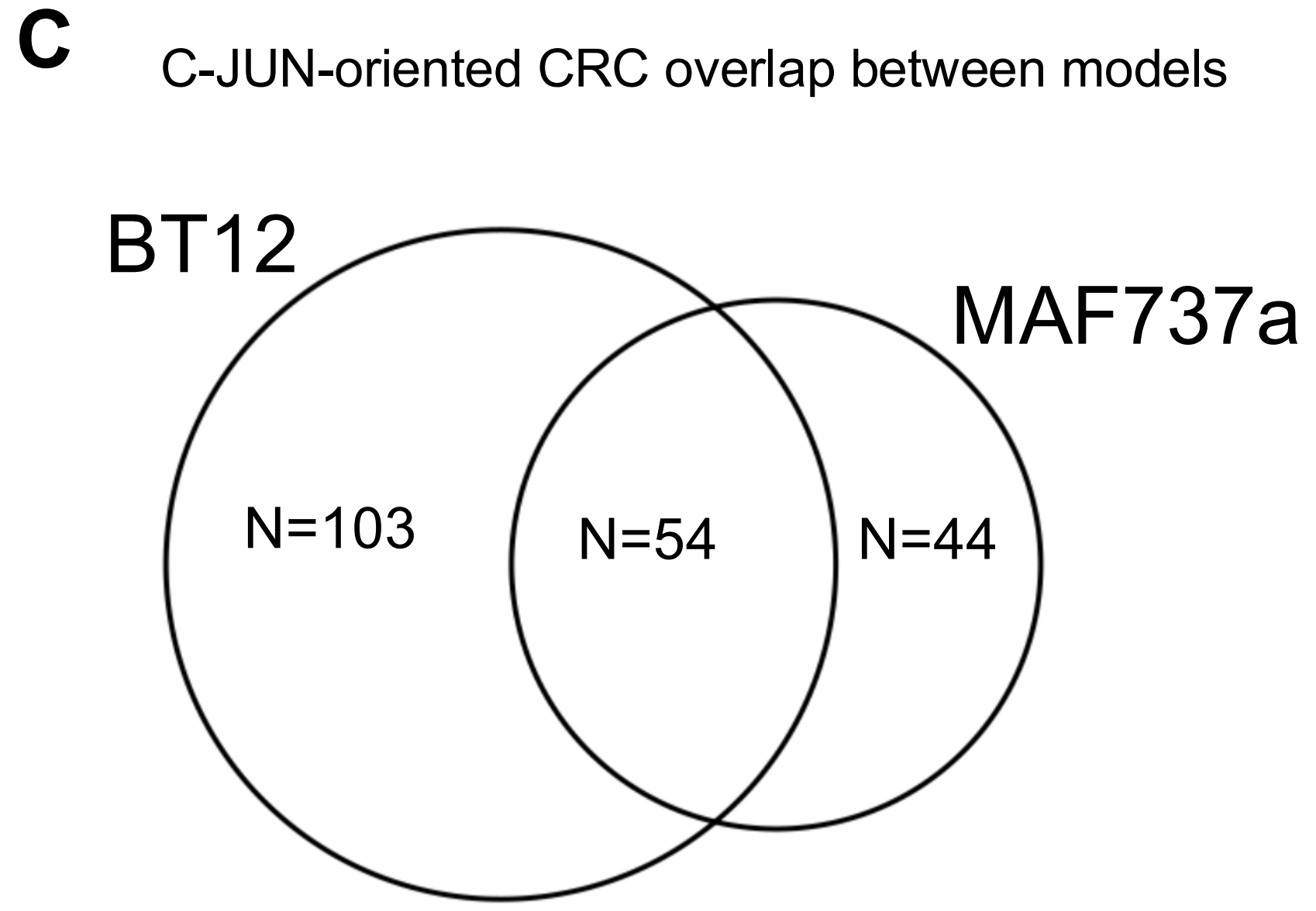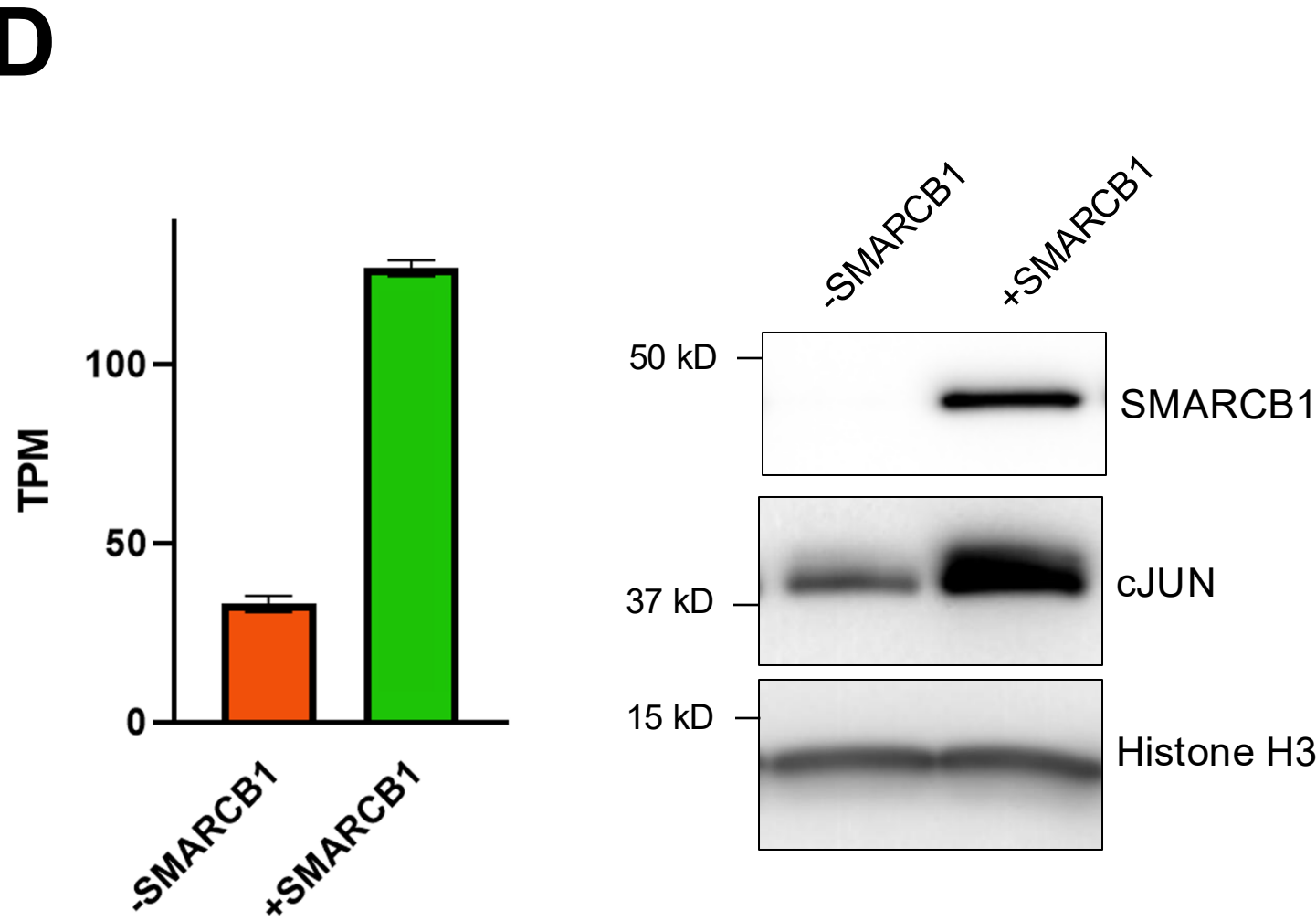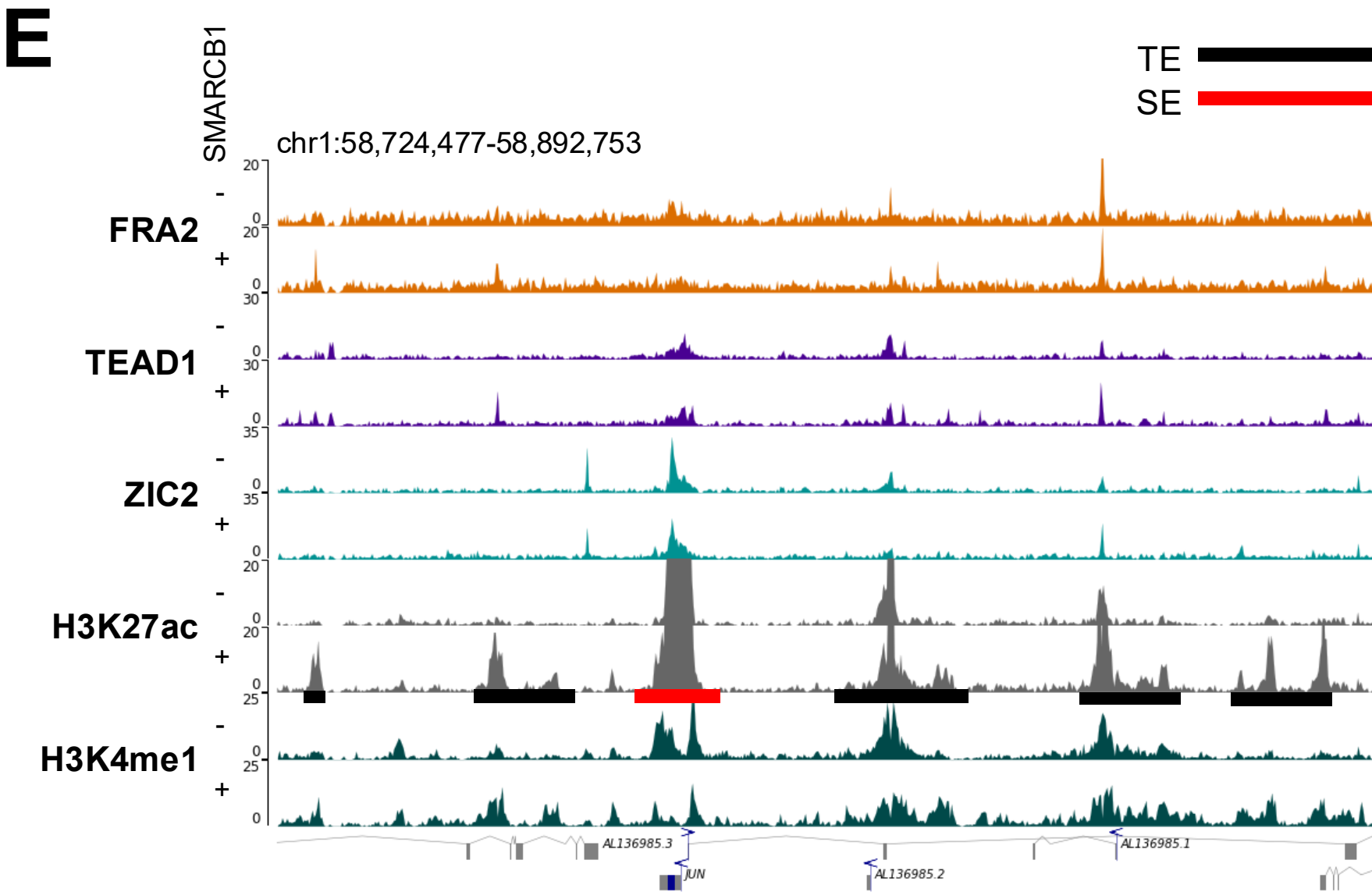

Supplement: noaf081_suppl_Supplementary_Figure_S8 [file noaf081_suppl_supplementary_figure_s8.pdf]

**A**

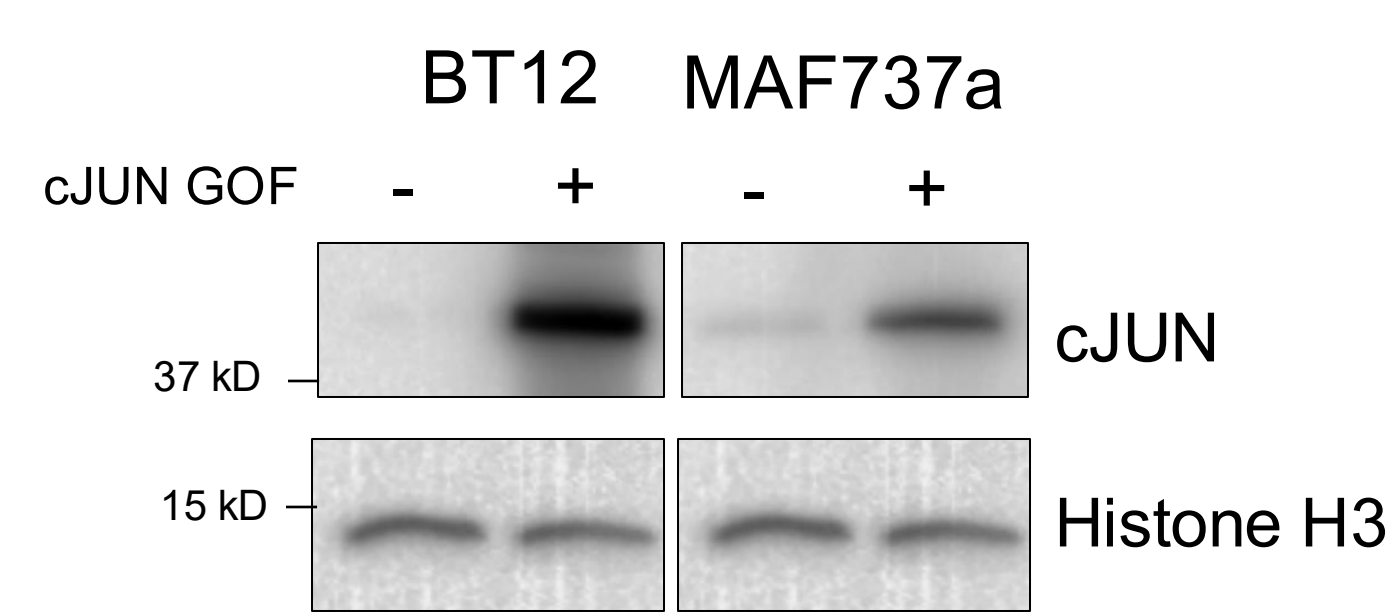

**B**

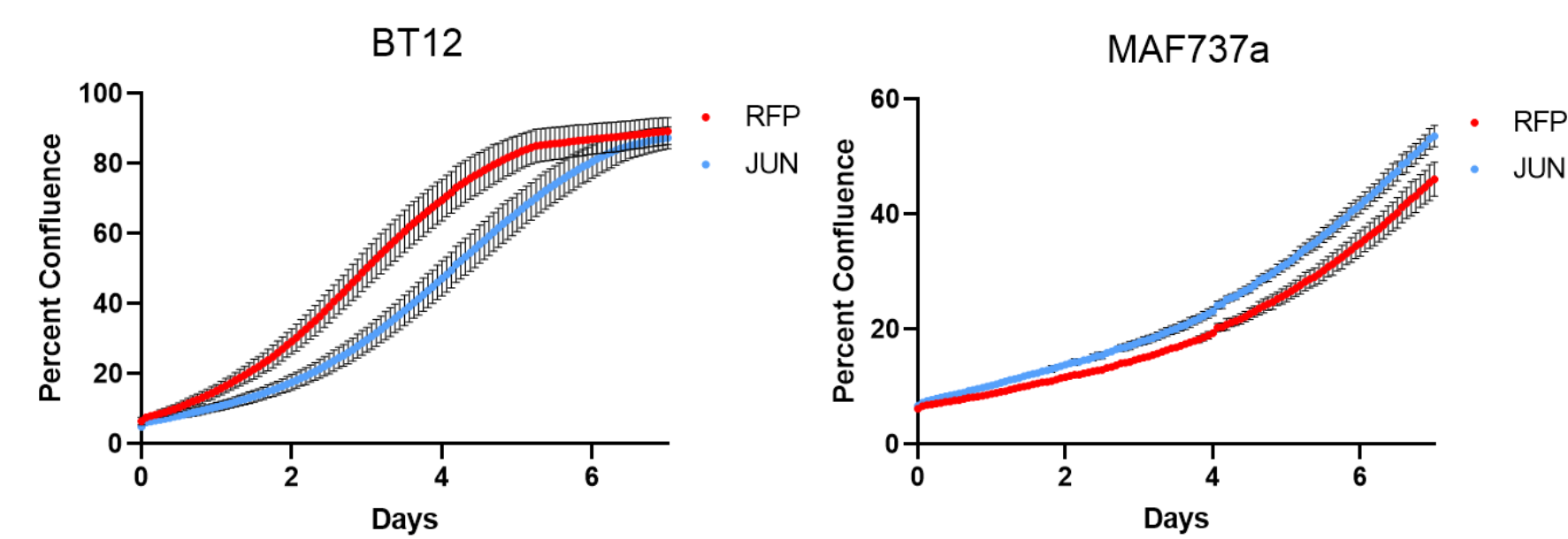

**C**

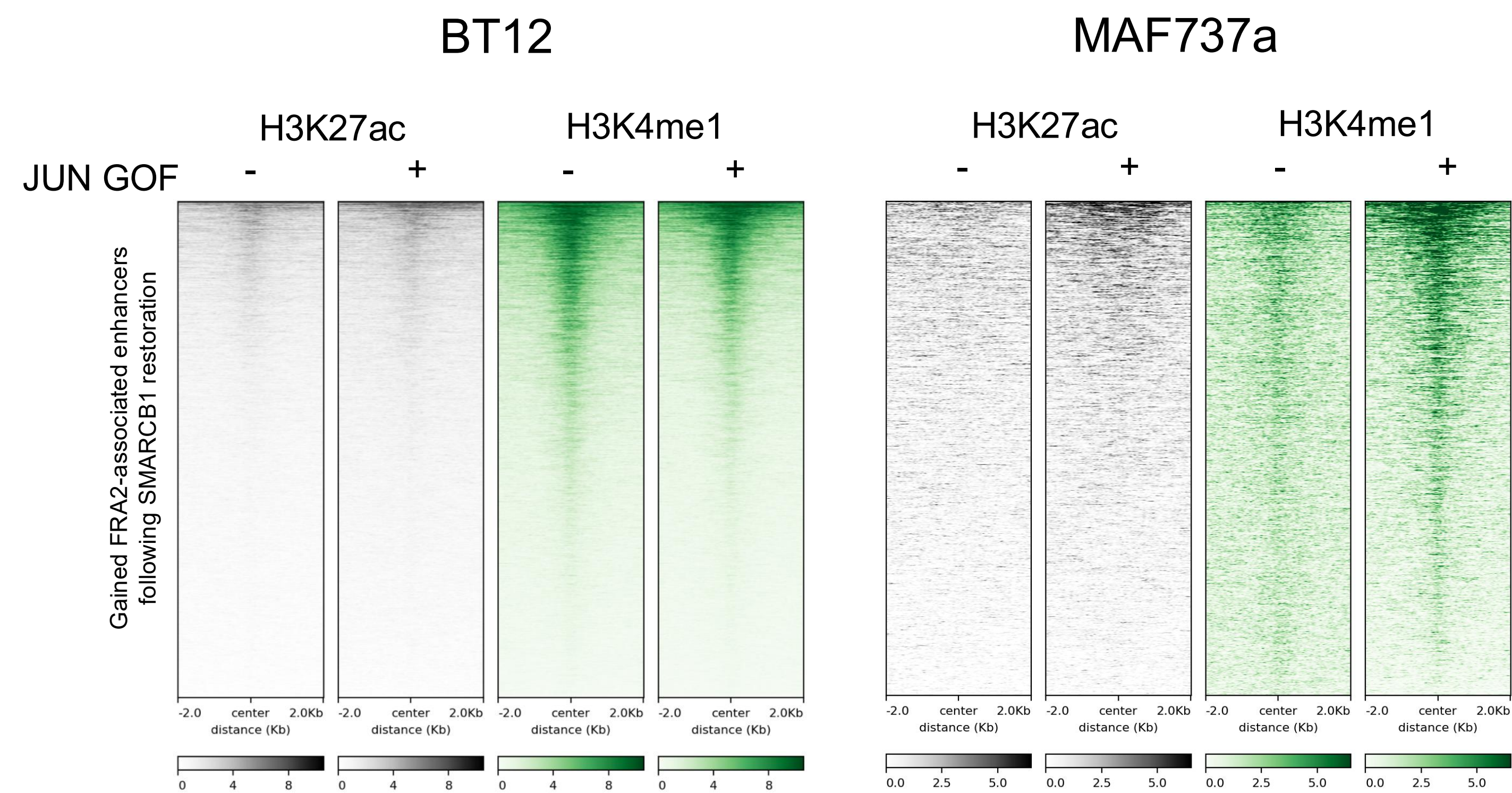

Supplement: noaf081_suppl_Supplementary_Figure_S9 [file noaf081_suppl_supplementary_figure_s9.pdf]

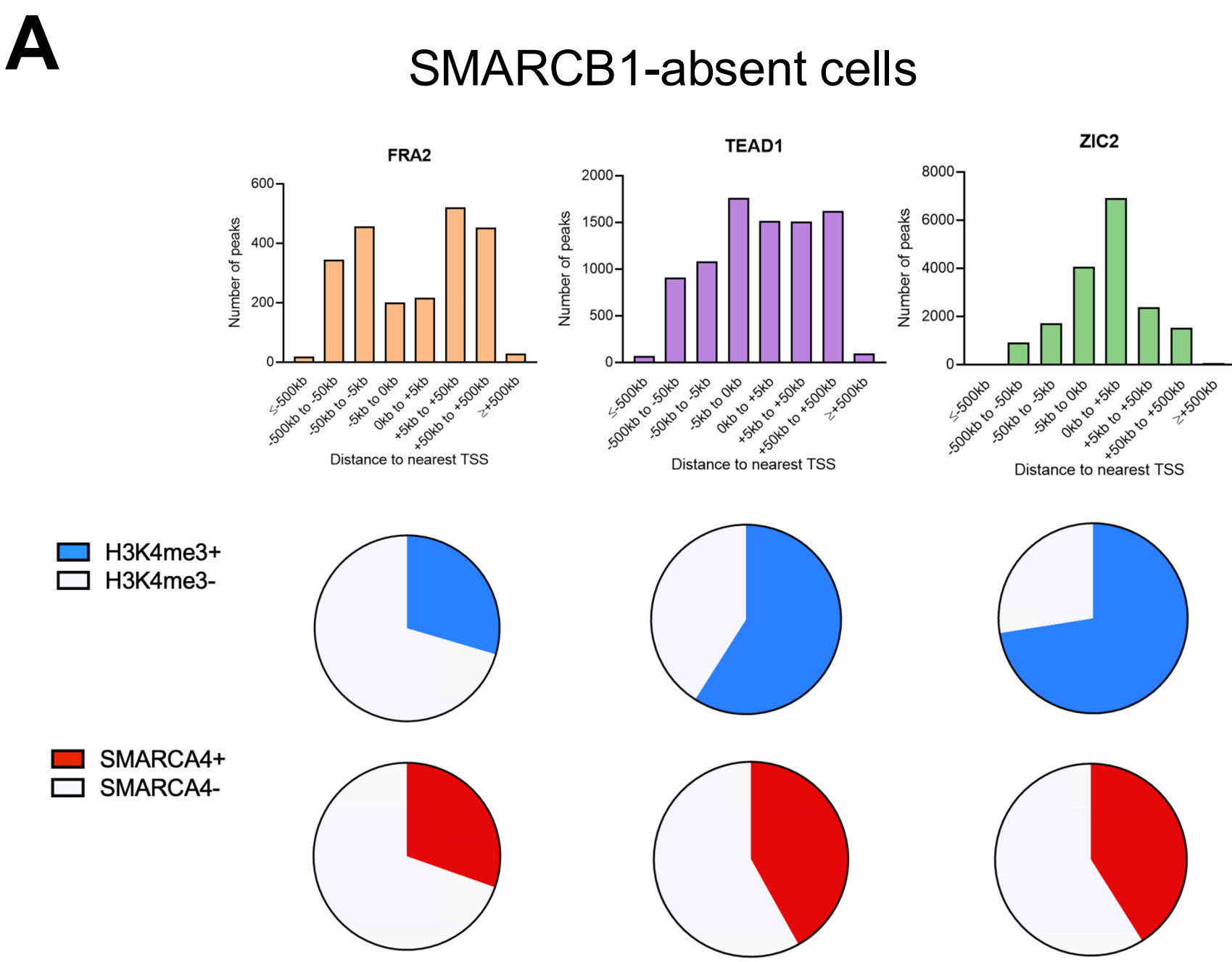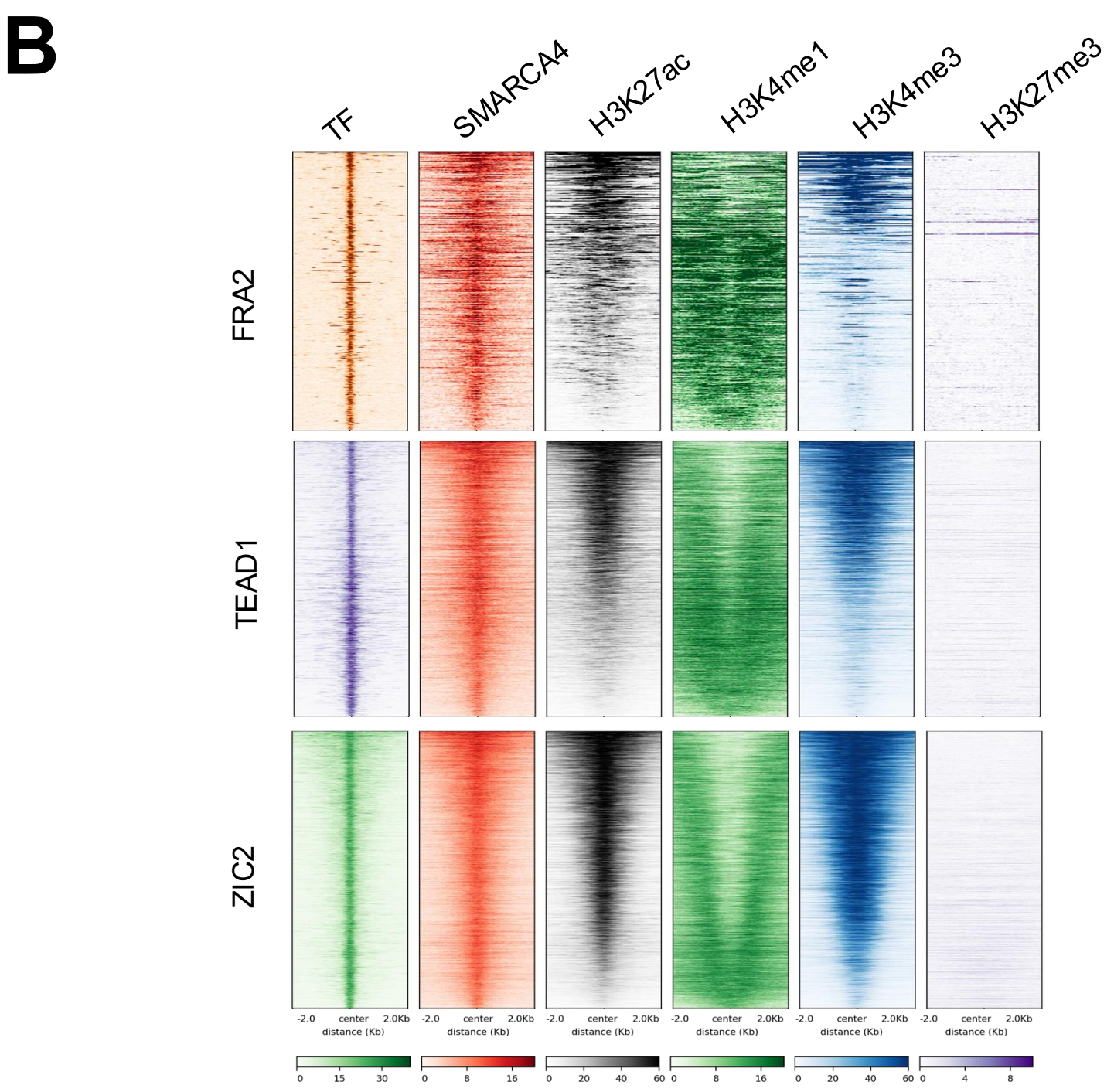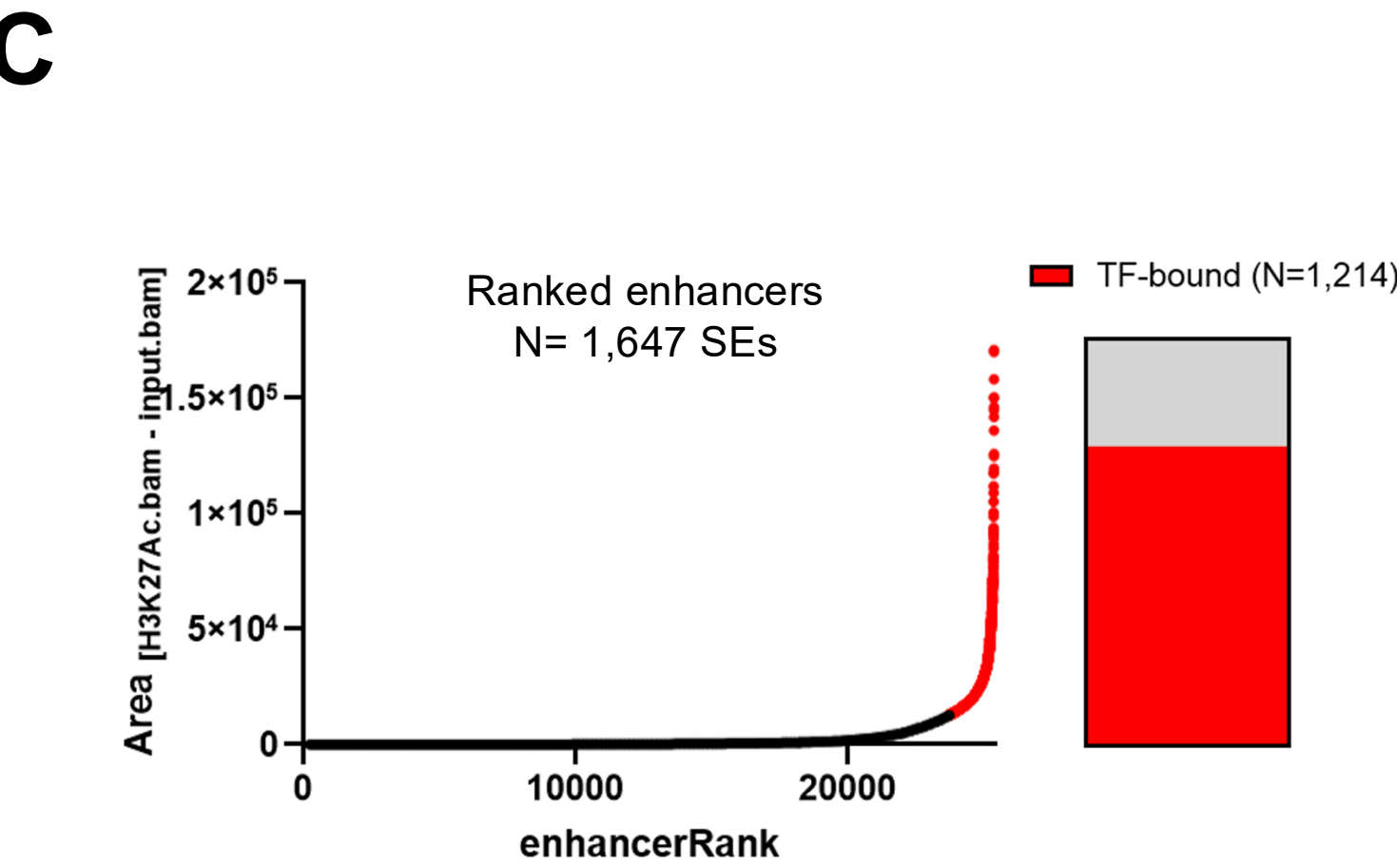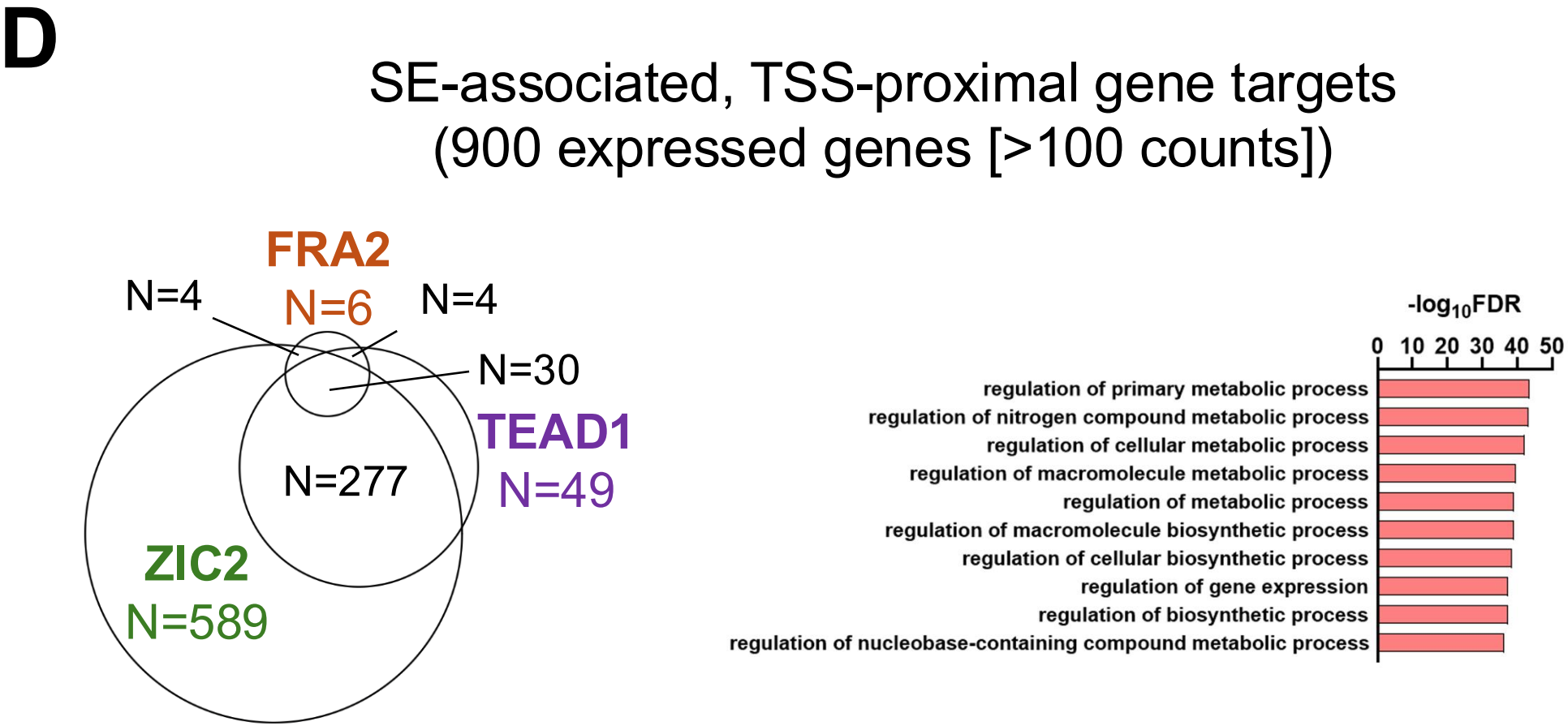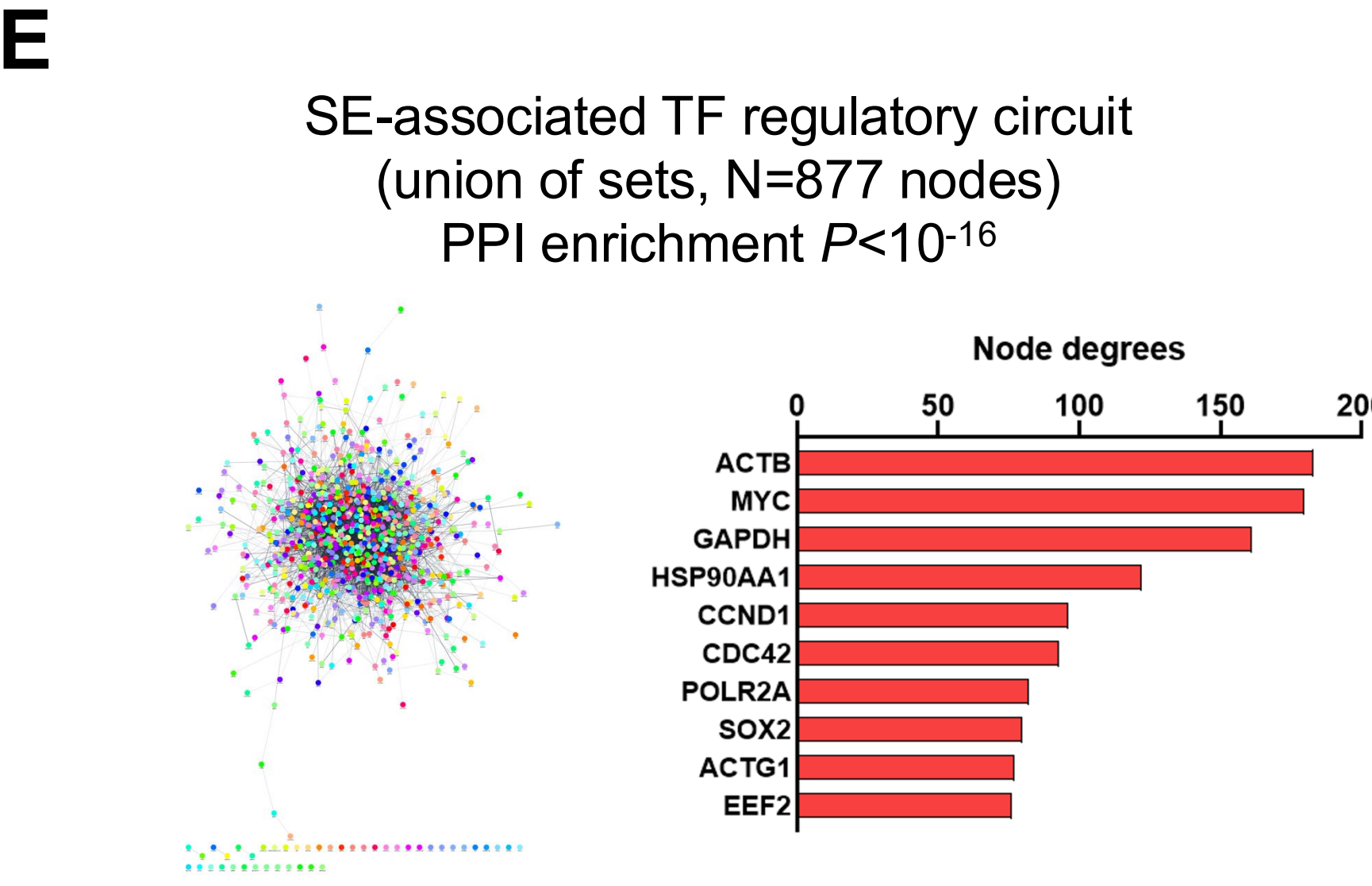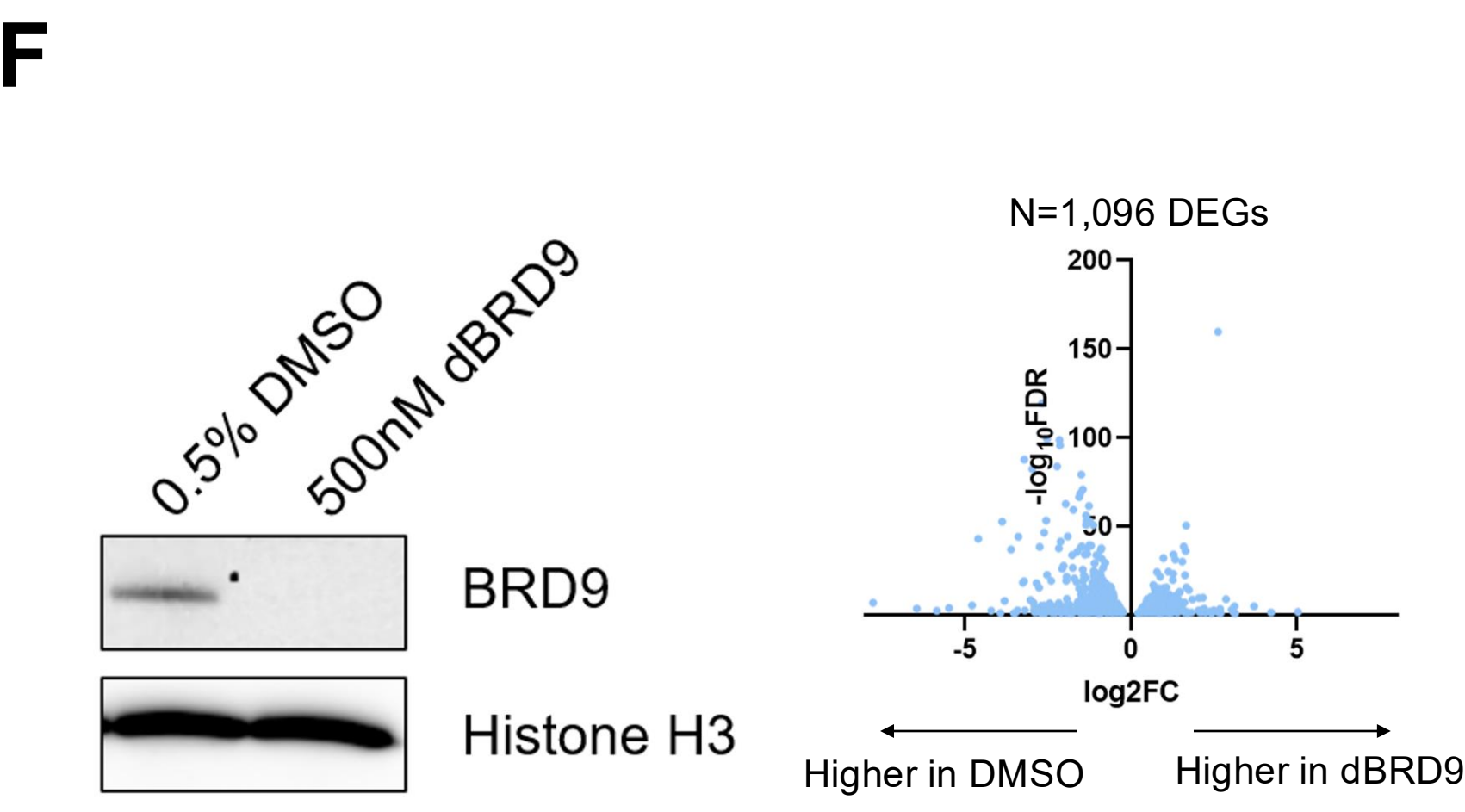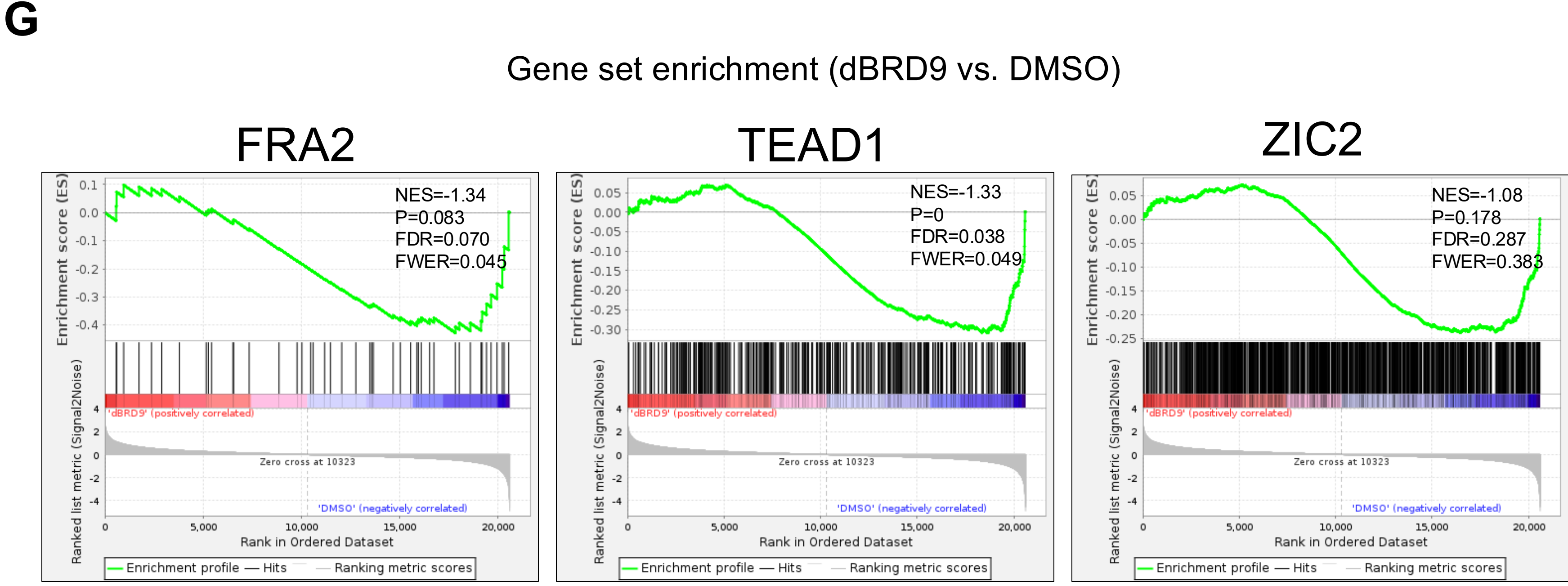

Supplement: noaf081_suppl_Supplementary_Figure_S10 [file noaf081_suppl_supplementary_figure_s10.pdf]

A

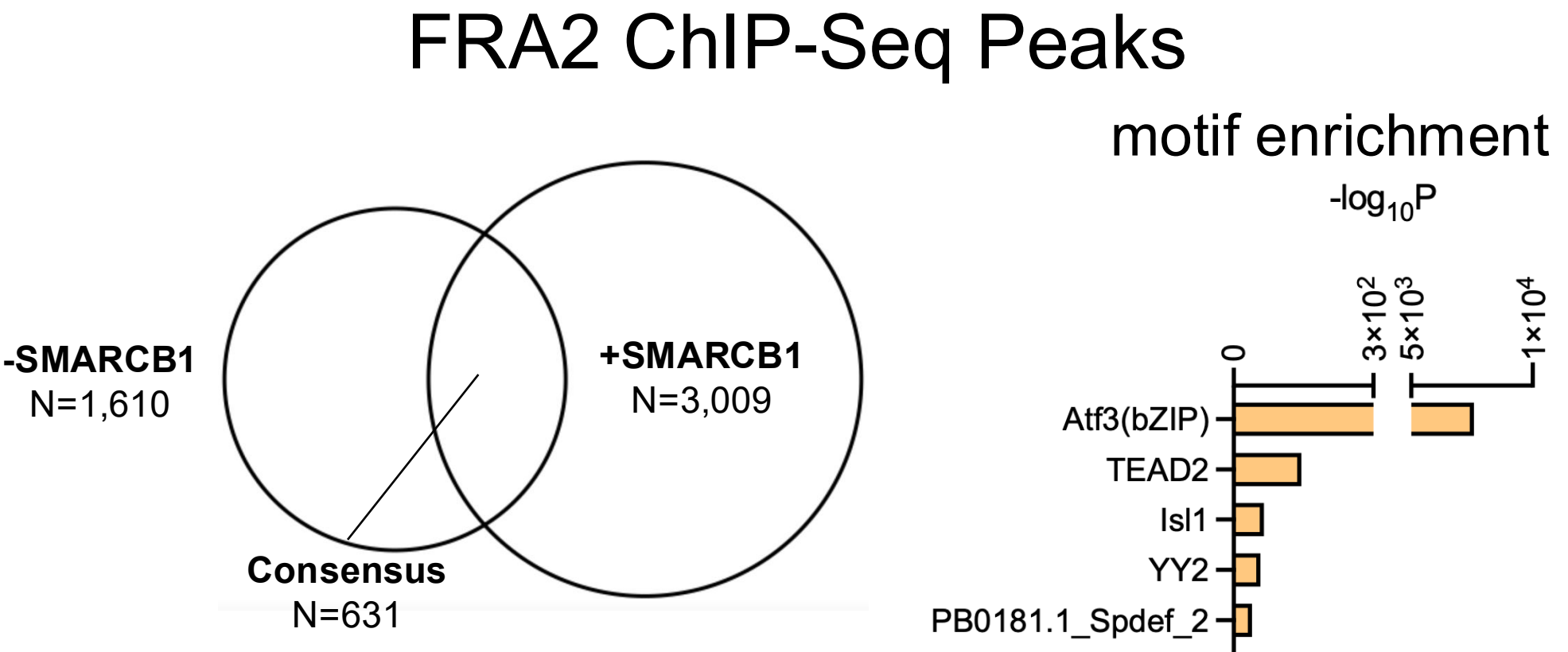

B

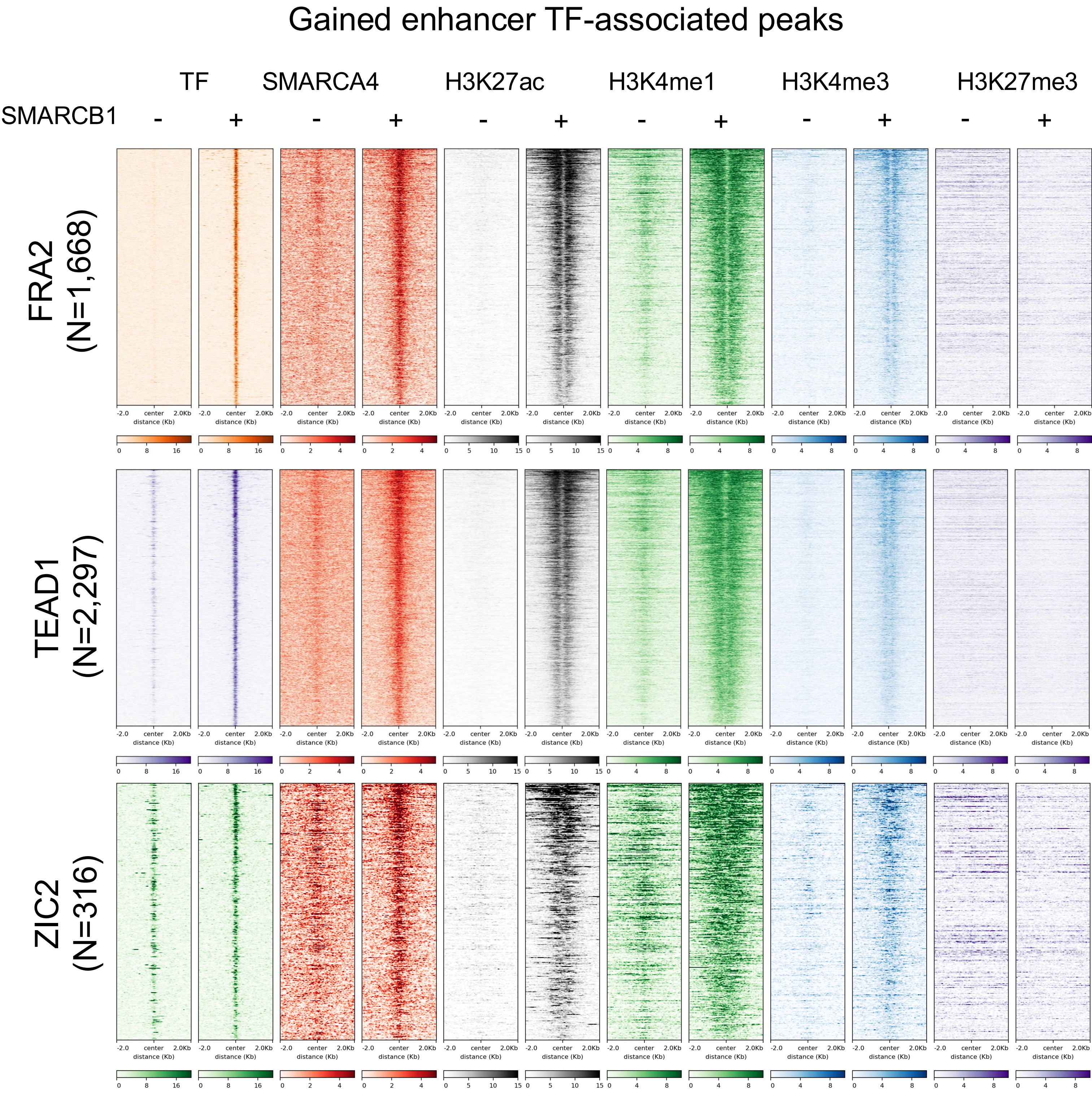

C

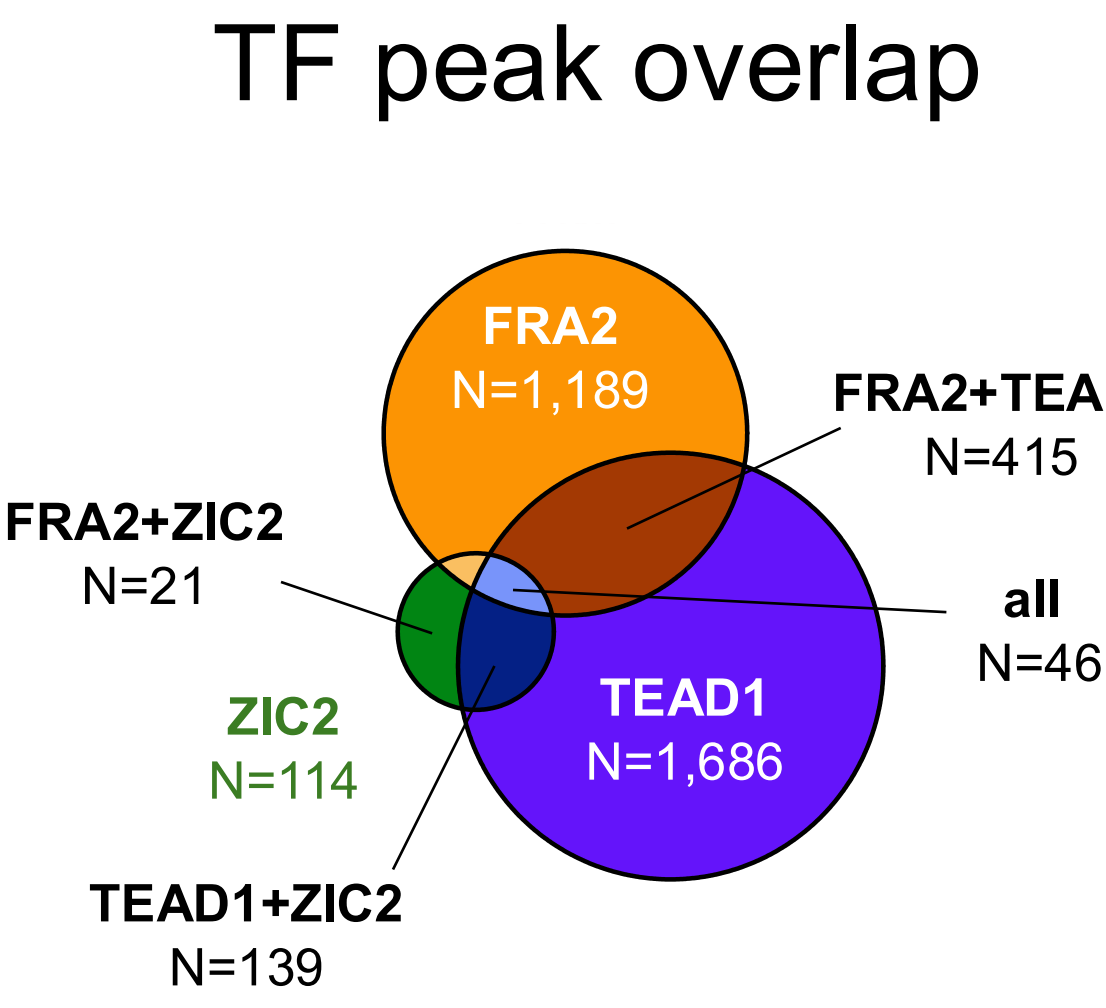

D

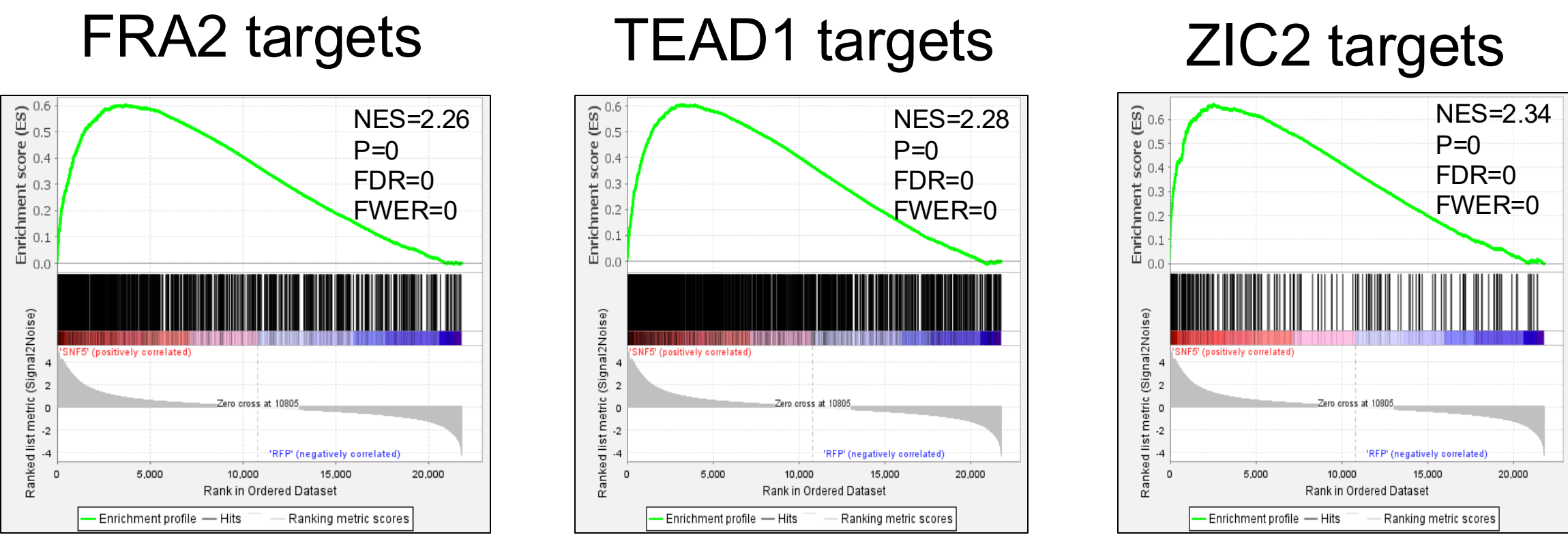

E

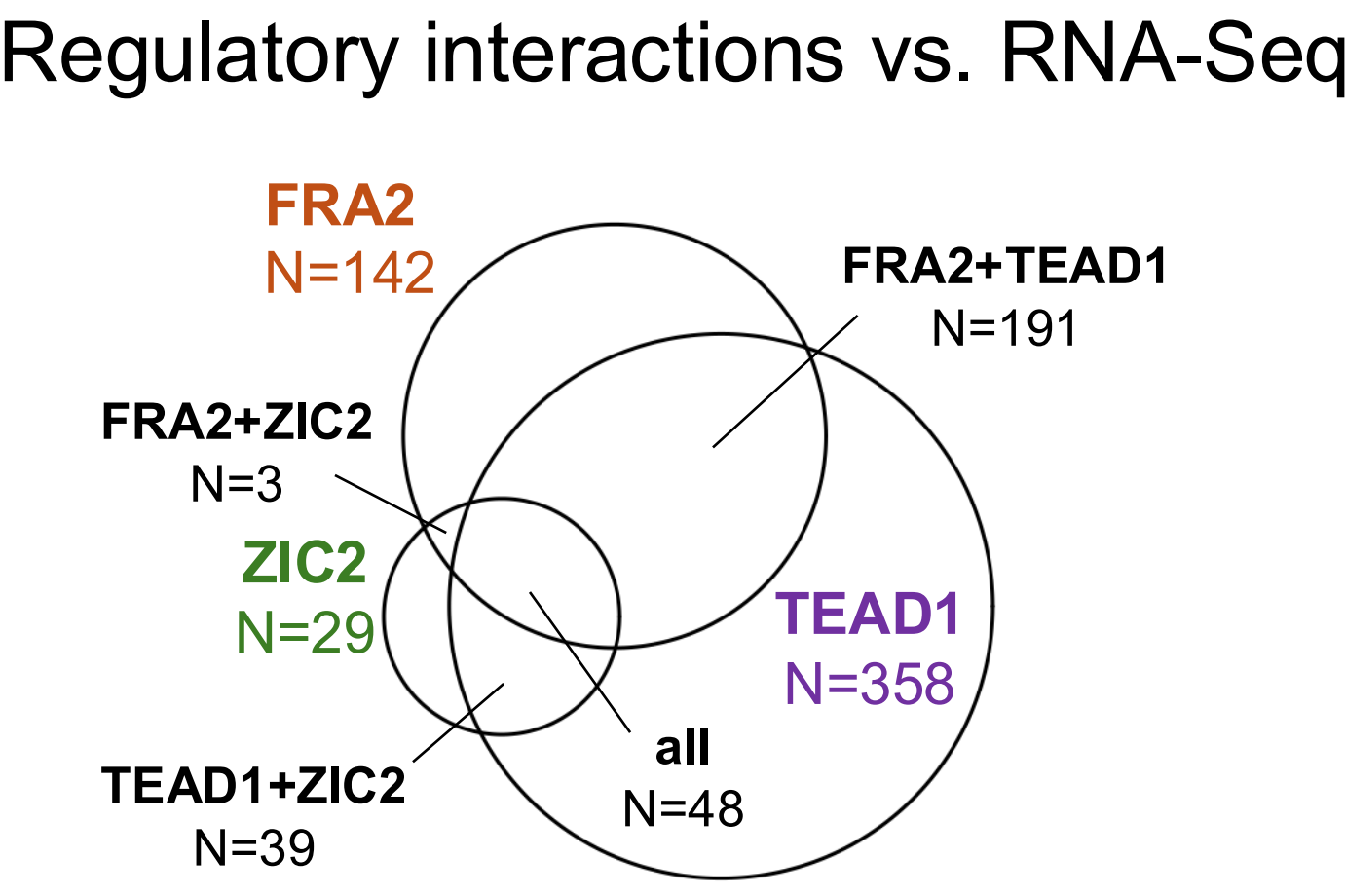

Supplement: noaf081_suppl_Supplementary_Figure_S4 [file noaf081_suppl_supplementary_figure_s4.pdf]
