# Supplementary material for: SWI/SNF complexes govern ontology-specific transcription factor function in MYC-subtype atypical teratoid rhabdoid tumor
Source: Neuro Oncol. 2025 Mar 23;27(9):2445–60. doi: 10.1093/neuonc/noaf081 (PMC12526124; doi:10.1093/neuonc/noaf081)

Epigenomic Associations of Transcription Factors (DJD29)

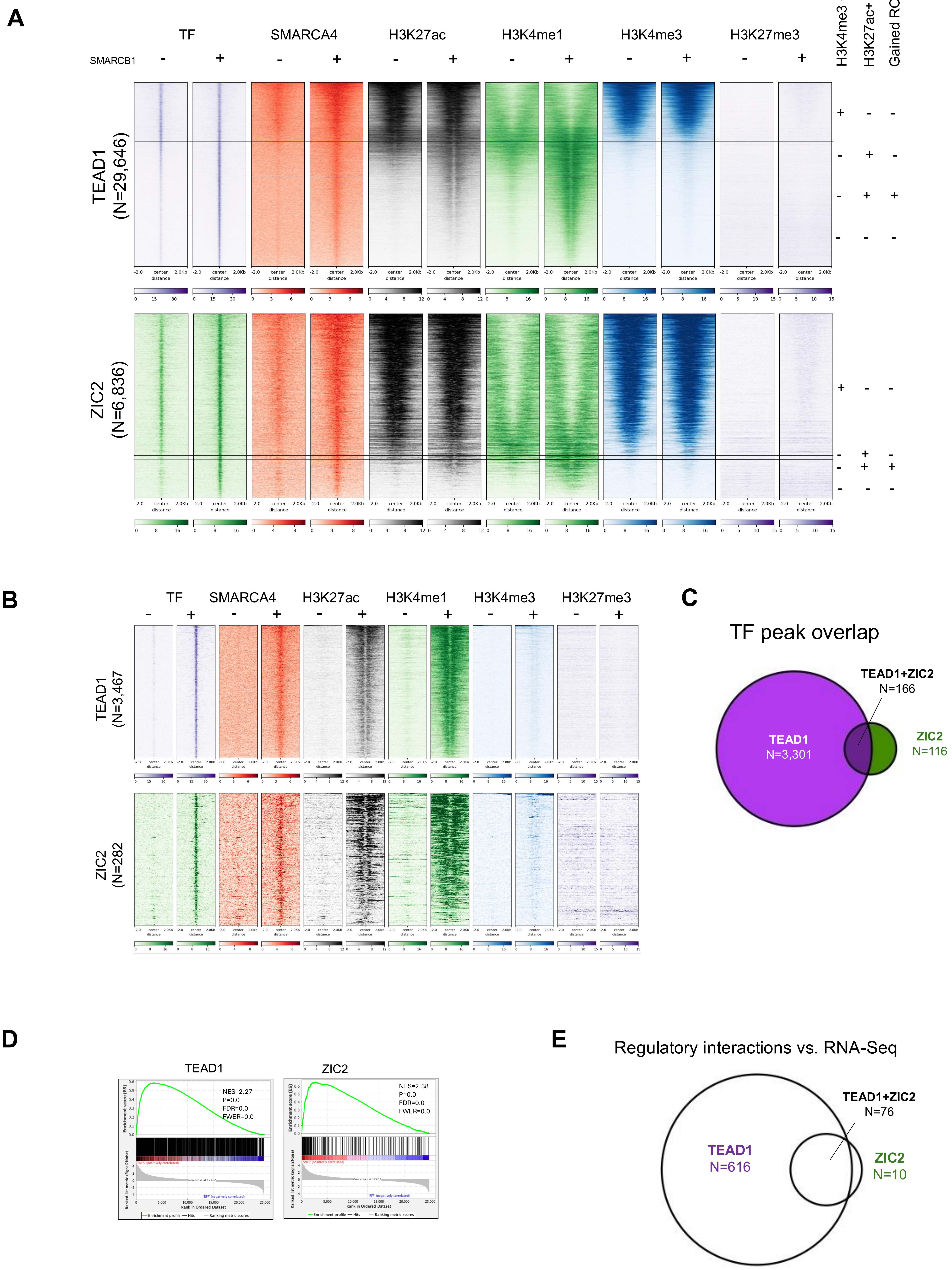

Supplement: noaf081_suppl_Supplementary_Figure_S7 [file noaf081_suppl_supplementary_figure_s7.pdf]
